# Runs of homozygosity and selection signature analyses reveal putative genomic regions for artificial selection in layer breeding

**DOI:** 10.1186/s12864-024-10551-4

**Published:** 2024-06-26

**Authors:** Xiaochang Li, Fangren Lan, Xiaoman Chen, Yiyuan Yan, Guangqi Li, Guiqin Wu, Congjiao Sun, Ning Yang

**Affiliations:** 1 State Key Laboratory of Animal Biotech Breeding, Frontiers Science Center for Molecular Design Breeding (MOE), and National Engineering Laboratory for Animal Breeding, China Agricultural University, Beijing, 100193 China; 2 Beijing Engineering Research Centre of Layer, Beijing, 101206 China

**Keywords:** Runs of homozygosity, Selective sweeps, GWAS, Layer breeding

## Abstract

**Background:**

The breeding of layers emphasizes the continual selection of egg-related traits, such as egg production, egg quality and eggshell, which enhance their productivity and meet the demand of market. As the breeding process continued, the genomic homozygosity of layers gradually increased, resulting in the emergence of runs of homozygosity (ROH). Therefore, ROH analysis can be used in conjunction with other methods to detect selection signatures and identify candidate genes associated with various important traits in layer breeding.

**Results:**

In this study, we generated whole-genome sequencing data from 686 hens in a Rhode Island Red population that had undergone fifteen consecutive generations of intensive artificial selection. We performed a genome-wide ROH analysis and utilized multiple methods to detect signatures of selection. A total of 141,720 ROH segments were discovered in whole population, and most of them (97.35%) were less than 3 Mb in length. Twenty-three ROH islands were identified, and they overlapped with some regions bearing selection signatures, which were detected by the De-correlated composite of multiple signals methods (DCMS). Sixty genes were discovered and functional annotation analysis revealed the possible roles of them in growth, development, immunity and signaling in layers. Additionally, two-tailed analyses including DCMS and ROH for 44 phenotypes of layers were conducted to find out the genomic differences between subgroups of top and bottom 10% phenotype of individuals. Combining the results of GWAS, we observed that regions significantly associated with traits also exhibited selection signatures between the high and low subgroups. We identified a region significantly associated with egg weight near the 25 Mb region of GGA 1, which exhibited selection signatures and has higher genomic homozygosity in the low egg weight subpopulation. This suggests that the region may be play a role in the decline in egg weight.

**Conclusions:**

In summary, through the combined analysis of ROH, selection signatures, and GWAS, we identified several genomic regions that associated with the production traits of layers, providing reference for the study of layer genome.

**Supplementary Information:**

The online version contains supplementary material available at 10.1186/s12864-024-10551-4.

## Background

Layer breeding places significant emphasis on the selection of egg-related traits such as egg number, weight, quality and appearance. Layer strains are subjected to long-term and intensive selection for the target traits, ensuring the development of efficient and high-performing commercial strains. Selection indexes including egg number (EN), egg weight (EW), quality and appearance traits have been used for decades in commercial breeding programs. It is commonly held that EW has a negative correlation with EN, prompting commercial breeders to prioritize higher EN and lower EW to meet the rising market demand for more eggs [1]. In addition, consumers seem to be focusing on the quality and appearance of eggs, which requires breeders to consider comprehensively the yield, quality, and appearance of eggs in order to meet consumers’ needs. With the advent of genomic selection (GS), it has become possible to investigate the genetic mechanism of important economic traits in greater depth [2]. The process of artificial selection has been accelerated by the application of GS, which has led to an increase in homozygosity in regions of the layer genome linked with egg-related traits [3].

Recently, runs of homozygosity (ROH) analysis has gained attention and is being increasingly adopted by researchers in the field to study population history and measure the degree of inbreeding [4–6]. ROH are continuous segments of a genome that are homozygous, meaning that the individual has inherited identical copies of genetic information from parents [7, 8]. ROHs can occur naturally in individuals as a result of inbreeding or heavy selection pressure and have hypothesized relevance with genes that influence disease susceptibility, cognitive ability, and production performance in individuals [6, 9, 10]. By utilizing ROH, scientists are able to better understand a population’s evolutionary history, inbreeding levels, and changes in genomic homozygosity in specific environments [11–13]. In agricultural research, ROH analysis has become an important tool for identifying genes and selection signatures that are associated with economic traits in livestock [3, 5, 14, 15]. Nevertheless, there are limitations in ROH research, mainly concerning the precise characterization of ROH in relation to the length and the number of loci [4, 16]. Presently, no definitive criteria have been established to determine the extent of ROH segments. Overall, the combination of ROH and selective sweep analysis can help us to gain a more comprehensive understanding of the genetic characteristics of different regions in the genome, and help to reveal the mechanisms associated with artificial selection and genetic variation to which chickens are subjected.

In this study, we used whole-genome sequencing (WGS) data to perform a genome-wide ROH study and multiple selective sweeps methods in order to detect and identify the putative regions subject to artificial selection for egg-related traits in a Rhode Island Red pure line with complete pedigree records. Rhode Island Red chickens are a widely used standard breed in the poultry industry due to their excellent egg production and quality. Specifically, we aimed to (i) investigate the distribution and frequency of ROH in this population, (ii) identify regions and genes within ROH islands that bear signals of selection across the whole population and annotate their functions, and (iii) combine the results of GWAS and selection signatures to analyze genomic differentiation and the differences of the incidence of SNP in ROH segments between subpopulations with high and low phenotype, to discovery genes or regions that may influence the phenotype. The traits used in this study include records at multiple time points of body weight (BW), egg number (EN), egg weight (EW), albumen height (AH), four kinds of eggshell color (ESCA, ESCB, ESCI and ESCL), eggshell strength (ESS), and glossiness (SINS) (Table 1). Our results will provide valuable insights into the genetic diversity and population structure of highly selected chicken populations and can inform breeding strategies to maintain and improve their productivity.

**Table 1:** Descriptive statistics of important chicken economical traits along with aging process

Traits	N	Mean	SD	CV (%)	Min	Max	h^2^
AH72	571	5.95	1.15	19%	2.4	9.4	0.44
AH80	490	5.28	1.55	29%	1	13.1	0.37
BW28	686	1924	141.44	7%	1482	2392	0.4
BW36	686	1947	174.15	9%	1575	2499	0.32
BW56	682	2065	202.59	10%	1374	2679	0.39
BW72	686	2111	228.96	11%	1271	2799	0.55
BW80	684	2190	237.87	11%	1365	3030	0.59
BWAFE	686	1760	120.21	7%	1335	2158	0.42
EN38	686	121	8.62	7%	100	144	0.25
EN48	686	186	9.90	5%	155	213	0.19
EN56	686	238	11.17	5%	196	268	0.16
EN72	686	339	17.29	5%	239	377	0.15
ESCA36	684	17.46	1.29	7%	12.61	21.5	0.29
ESCA56	674	17.40	1.77	10%	2.17	23.5	0.19
ESCA72	659	17.47	1.96	11%	5.11	22.18	0.18
ESCA80	658	16.86	1.68	10%	10.11	21.79	0.48
ESCB36	684	28.61	1.30	5%	23.17	32.55	0.48
ESCB56	674	28.94	1.70	6%	12.39	32.52	0.35
ESCB72	659	29.04	2.06	7%	17.86	32.43	0.2
ESCB80	658	28.26	1.77	6%	16.81	32.32	0.29
ESCI36	684	12.50	4.29	34%	-0.43	28.46	0.33
ESCI56	674	16.37	5.54	34%	1.75	63.83	0.28
ESCI72	659	14.69	6.45	44%	1.35	49.37	0.26
ESCI80	658	16.11	5.81	36%	-0.57	38.72	0.32
ESCL36	684	58.57	2.88	5%	47.39	68.12	0.33
ESCL56	674	62.70	3.11	5%	52.20	78.39	0.36
ESCL72	659	61.20	3.37	6%	51.57	76.35	0.36
ESCL80	658	61.23	3.45	6%	51.04	72.55	0.33
ESS36	682	3.49	0.64	18%	1.05	5.40	0.39
ESS56	673	3.48	0.57	16%	1.48	5.46	0.25
ESS72	655	2.91	0.63	22%	1.47	5.22	0.33
ESS80	652	3.31	0.62	19%	1.03	5.18	0.39
EW28	685	56.22	3.53	6%	36.3	68	0.37
EW36	686	57.62	3.81	7%	44.6	68.6	0.45
EW56	675	60.78	4.23	7%	42.5	74.9	0.41
EW72	664	60.67	4.27	7%	46.6	76.6	0.45
EW80	654	61.55	4.44	7%	49.1	74.9	0.52
EWAFE	684	43.18	7.06	16%	21.4	88	0.19
SINS36	683	3.16	0.59	19%	1.9	5.6	0.11
SINS56	673	2.45	0.49	20%	1.4	4.8	0.18
SINS72	659	2.36	0.45	19%	1.3	4	0.15
SINS80	658	2.43	0.56	23%	1.43	4.6	0.18

*Abbreviations*: *AH* Albumen height (mm), *BW* Body weight (g), *AFE* Age at first egg, *EN* Egg number, *ESC* Eggshell color, *ESS* Eggshell strength, *EW* Egg weight (g), *SINS* Eggshell gloss. The number following the trait indicates the age of week, *N* Number of samples, *Mean* Average value, *SD* Standard deviation, *CV (%)* Coefficient of variation, *Min* Minimum value, *Max* Maximum value, *h*^*2*^ Heritability

## Results

### Characteristics of ROH and F(ROH)

**Fig. 1:**
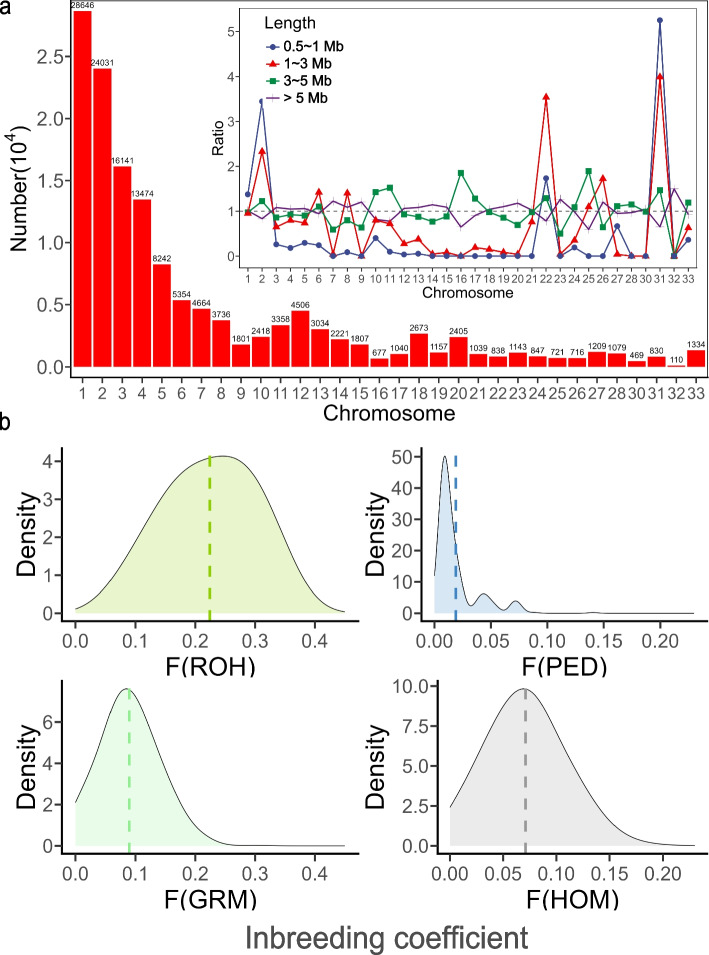
Descriptive graphics of runs of homozygosity (ROH) in Rhode Island Red chickens. **a** The distribution of ROH on chromosomes and the proportion of ROH for different lengths; **b** Inbreeding coefficients calculated by four methods: F(ROH) by PLINK, F(GRM) by G matrix in GCTA, F(PED) by pedigree in CFC, and F(HOM) by homozygosity in PLINK

A total of 141,720 ROH segments were identified in this population with an average length of 1.043 Mb, an average of 206.6 segments per individual, and 4,085 SNPs per segment. The largest ROH segment was 10.17 Mb in length and located between 117.39 and 127.56 Mb on GGA 2, containing 55,091 SNPs. Most of the ROH segments were located on chromosomes 1–4 (including 82,292 ROHs), accounting for 58.07% of the total (Fig. 1a). To distinguish ROH segments of different lengths, we artificially set three thresholds (1 Mb, 3 Mb and 5 Mb) to categorize them into four classes, and found that the proportions of these four ROH categories on each chromosome were not identical to the proportions in the entire genome. For instance, on large chromosomes, the proportions of each type were comparable; however, on small chromosomes, there tended to be a larger proportion of ROH segments longer than 3 Mb (Fig. 1a).

When comparing inbreeding coefficients, F(ROH), with a mean of 0.224, was much higher than other calculations (F(PED): 0.019, F(GRM): 0.088, F(HOM): 0.070, Fig. 1b). And due to the different SNPs used, it was also observed that F(ROH) had low correlation coefficients (0.097 ~ 0.331) with F(GRM) and F(HOM), which used independent SNPs obtained by linkage disequilibrium (LD) pruning rather than all SNPs on genome. In addition, low Pearson correlation coefficient (0.097 ~ 0.348) between F(PED) and F(ROH), F(GRM), and F(HOM) were observed (see Additional file 1 Table S1), reflecting the fact that there might be relatively large differences between pedigree and genomic data for judging the degree of inbreeding.

**Fig. 2:**
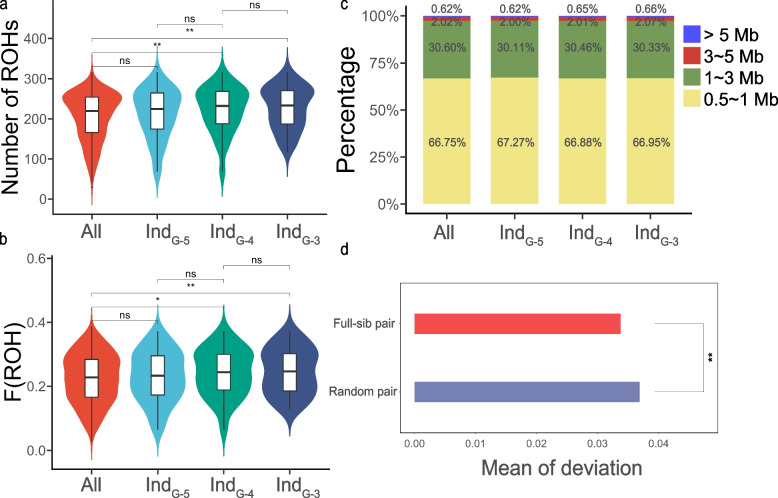
The degree of inbreeding measured by runs of homozygosity (ROH). “Ind_G-n_” represents individuals whose parents’ common ancestor appeared “n.^th^” generations ago. **a** The number of ROH in samples with different degrees of inbreeding; **b** F(ROH) of samples with different degrees of inbreeding; **c** The percentage of total ROH within each ROH length category; **d** The difference between two full siblings’ F(ROH) and random pairs. *p < 0.05, **p < 0.01, ***p < 0.001, ****p < 0.0001

In the population, we also found some individuals whose parents shared a common ancestor within the six-generation pedigree. Consequently, we formed subsets of individuals whose parents had a common ancestor from the same generation. Moreover, we posited that if the parents’ common ancestor appeared in later generations, the individual would exhibit a higher degree of inbreeding. We found that individuals whose parents shared a common ancestor in last four generations had a significantly higher number of ROH (223.57 ~ 228.14, Fig. 2a, *p* value < 0.01) and F(ROH) (0.242 ~ 0.247, Fig. 2b, *p* value < 0.01) than the population mean (number of ROH: 206.59; F(ROH): 0.224), and the greater the degree of inbreeding was, the larger the difference. However, this difference was no longer significant when the common ancestor appeared five generations earlier, suggesting that ROH was adept at reflecting recent inbreeding events. Similarly, individuals whose parents shared a common ancestor in last four generations had a higher proportion of long ROH (0.65 ~ 0.66%) than the population mean (0.62%) (Fig. 2c). These results confirmed that the use of F(ROH) to characterize the degree of inbreeding was relatively reliable in this Rhode Island Red pure line. Additionally, we observed that the deviation of F(ROH) between full siblings (mean = 0.032) was lower than the mean deviation between a random pair (0.037, *p* value = 0.025), indicating a relatively stable and common occurrence of ROH segments (Fig. 2d). Overall, ROH analysis is perfectly suited for studying inbreeding and selection in this group.

### ROH islands and De-correlated composite of multiple signals

**Fig. 3:**
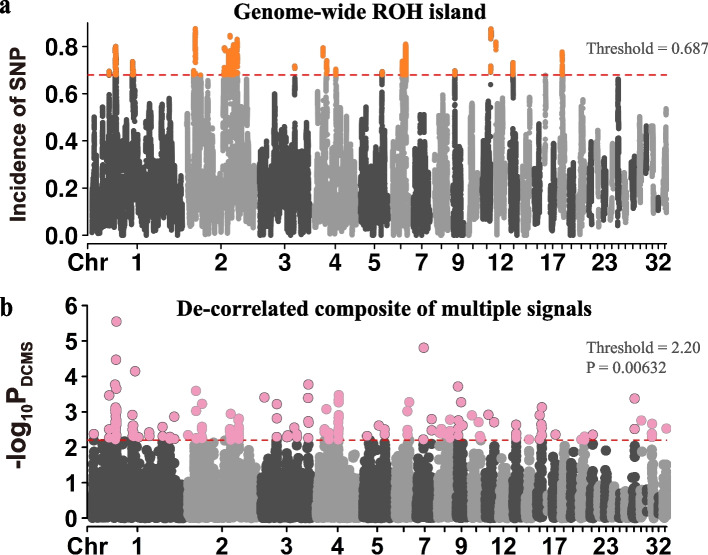
Manhattan plot of the incidence of SNPs in ROH and selective sweeps detected by DCMS. **a** Whole genome-wide incidence of SNPs in ROH, which indicates the ROH islands in the population.; **b** Selective sweeps detected by DCMS on whole genome

For the whole population, we initially identified 23 ROH islands based on regions containing the top 1% most frequent loci in the ROH segments (Fig. 3a, threshold = 0.687) and then annotated the known QTL regions that overlapped with them (Table 2). Most ROH islands were distributed on GGA 1–6 (number: 18, accounting for 78.26%), and the average length of ROH islands was 1.13 Mb, containing a mean of 2058.6 SNPs, which revealed that ROH islands might be more common in regions with low SNP density (the average of SNP density of ROH segments was 4,085, as mentioned above). The average of minor allele frequency (MAF) of SNPs (*n* = 47,348) in ROH islands was 0.083, much lower than the average of all SNPs (0.243). In addition, the average incidence of SNPs in ROH segments for all ROH island regions was 73.41%, ranging from 68.68% to 84.81%, much higher than the average of all SNPs (17.70%).

**Table 2:** ROH islands overlapped with reported QTLs

Position	Region average of incidence of SNP in ROH (%)	Number of SNP	Overlapped QTL trait(s)
1:36,940,128–37194844	0.69	944	-
1:50,146,153–52750619	0.74	4798	Feed conversion ratio / Egg number / Abdominal fat percentage / Body weight
1:88,042,802–88630511	0.72	1079	Feed intake / Body weight
1:89,771,664–90,206,011	0.69	734	Thigh muscle pH
2:14,947,422–15,310,050	0.69	2008	Chest width
2:18,142,793–19,271,979	0.82	1385	Abdominal fat weight / Feed conversion ratio
2:80,825,999–84272009	0.72	207	Feather pecking / Breast muscle pH / Eggshell weight / Albumen height
2:91,329,911–95,615,858	0.73	6523	Body weight / Abdominal fat percentage
2:100,871,490–103316229	0.73	7673	Feed conversion ratio / Body weight
2:110,056,725–111200505	0.79	3975	Breast muscle pH / Body temperature
3:77,258,932–77,530,838	0.71	187	Shank circumference / Feather pecking / Eggshell color
4:18,649,833–19,274,577	0.78	200	-
4:26,151,029–27447735	0.72	3225	Body depth
4:46,619,643–46,991,705	0.69	2035	Egg weight
5:46,642,314–47,290,496	0.69	1094	Antibody titer to IBV / Body weight
6:21,389,648–22,209,999	0.71	697	Eggshell stiffness / Eggshell cuticle coverage / Body weight / Breast muscle pH
6:26,246,593–26,431,590	0.69	593	-
6:28,457,718–30,108,964	0.76	1461	Feather pecking / Feed conversion ratio / Body weight
9:4,539,012–4871440	0.69	621	Feed conversion ratio
11:18,750,256–20156406	0.85	999	Body weight / Egg production rate / Breast muscle weight / Body weight
12:31,451–610,718	0.81	443	-
13:7,808,825–8225656	0.71	2193	Feather pecking / Feed conversion ratio / Breast muscle weight
18:5,622,977–6,399,812	0.74	4274	Feather pecking / Body weight / Abdominal fat weight

The De-correlated composite of multiple signals (DCMS) consolidates three within-population statistics into a comprehensive score, including nucleotide diversity (Pi), Tajima’s D, and integrated Haplotype Score (iHS). Upon computing the DCMS statistic, we fitted the *p*-values to a normal distribution to identify candidate sweep regions by evaluating the empirical distribution’s top 1% (Fig. 3b, threshold = 2.20). This approach delineated a total of 176 candidate regions, with 127 of these regions situated on GGA 1–6 (72.16%). Out of 23 detected ROH islands, 12 coincided with these candidate regions, while 4 lay within 1 Mb proximity. Consequently, we identified 59 regions of overlap between ROH islands and selection signatures, corresponding to 60 genes (Table 3). Functional annotation of these identified genes suggested that they contributed to encompassing growth, immunity, disease resistance, cardiovascular and neurological functions in layers (Table 4). Notably, a number of pathways indicate that certain genes influence mRNA synthesis and metabolism. Moreover, the LARGE1, SLC18A2, and CACNB2 genes interact with various neural signal transduction pathways, while the LYN and LGALS2 genes are implicated in the regulation of immune responses.

**Table 3:** Genes located in overlapping regions of ROH islands and selective sweeps

Gene	Gene Name	Position	Description
ENSGALG00000012050	TNRC6B	1:50,120,269–50164133	trinucleotide repeat containing 6B
ENSGALG00000012236	DMC1	1:50,865,020–50876251	DNA meiotic recombinase 1
ENSGALG00000037360	DDX17	1:50,877,655–50894778	DEAD-box helicase 17
ENSGALG00000032313	KDELR3	1:50,897,173–50902317	KDEL endoplasmic reticulum protein retention receptor 3
ENSGALG00000012254	KCNJ4	1:50,905,908–50917205	Potassium inwardly rectifying channel subfamily J member 4
ENSGALG00000053778	LOC101747255	1:50,909,163–50911116	serologically defined colon cancer antigen 3 homolog
ENSGALG00000032257	CSNK1E	1:50,927,995–50948440	casein kinase 1 epsilon
ENSGALG00000012285	BAIAP2L2	1:50,999,516–51007562	BAI1 associated protein 2 like 2
ENSGALG00000036897	SLC16A8	1:51,008,968–51016634	solute carrier family 16 member 8
ENSGALG00000041823	PICK1	1:51,019,143–51030492	protein interacting with PRKCA 1
ENSGALG00000012290	SOX10	1:51,055,215–51064410	SRY-box 10
ENSGALG00000012291	POLR2F	1:51,067,332–51070945	RNA polymerase II subunit F
ENSGALG00000012293	C22orf23	1:51,071,012–51075308	chromosome 1 C22orf23 homolog
ENSGALG00000012265	MICALL1	1:51,075,320–51093611	MICAL like 1
ENSGALG00000012299	ANKRD54	1:51,089,196–51112478	ankyrin repeat domain 54
ENSGALG00000012296	EIF3L	1:51,097,092–51106741	eukaryotic translation initiation factor 3 subunit L
ENSGALG00000012307	GALR3	1:51,117,014–51118848	galanin receptor 3
ENSGALG00000019312	LOC693258	1:51,121,427–51,122,146	noggin 4
ENSGALG00000012312	GCAT	1:51,123,081–51128130	glycine C-acetyltransferase
ENSGALG00000012410	NOL12	1:51,151,276–51,155,431	nucleolar protein 12
ENSGALG00000012416	LOC100858460	1:51,155,529–51,162,122	arf-GAP with dual PH domain-containing protein 1-like
ENSGALG00000012419	UTS2RL	1:51,162,834–51,165,452	urotensin-2 receptor-like
ENSGALG00000012420	CG-1B	1:51,166,508–51169768	galectin 1
ENSGALG00000023131	PDXP	1:51,181,690–51185095	pyridoxal phosphatase
ENSGALG00000012422	SH3BP1	1:51,186,462–51,193,519	SH3 domain binding protein 1
ENSGALG00000039658	GGA1	1:51,196,551–51,205,052	golgi associated, gamma adaptin ear containing, ARF binding protein 1
ENSGALG00000003213	LGALS2	1:51,206,946–51218711	galectin 2
ENSGALG00000038556	CDC42EP1	1:51,221,544–51,223,454	CDC42 effector protein 1
ENSGALG00000042365	CARD10	1:51,238,459–51,251,963	caspase recruitment domain family member 10
ENSGALG00000012442	MFNG	1:51,255,614–51,276,612	MFNG O-fucosylpeptide 3-beta-N-acetylglucosaminyltransferase
ENSGALG00000012446	ELFN2	1:51,333,318–51,335,744	extracellular leucine rich repeat and fibronectin type III domain containing 2
ENSGALG00000012454	CYTH4	1:51,385,635–51,405,059	cytohesin 4
ENSGALG00000012522	PVALB	1:51,608,164–51618692	parvalbumin
ENSGALG00000042990	IFT27	1:51,627,090–51635098	intraflagellar transport 27
ENSGALG00000012540	RBFOX2	1:51,917,777–51,989,848	RNA binding protein, fox-1 homolog 2
ENSGALG00000012541	MB	1:52,004,040–52007757	myoglobin
ENSGALG00000012542	RASD2	1:52,034,440–52042836	RASD family member 2
ENSGALG00000012559	LARGE1	1:52,678,868–52,954,337	LARGE xylosyl- and glucuronyltransferase 1
ENSGALG00000054689	LOC101752020	1:88,465,698–88,466,831	inositol 1,4,5-trisphosphate receptor-interacting protein-like 1-like
ENSGALG00000037769	NEBL	2:18,124,237–18,368,109	nebulette
ENSGALG00000007956	PLXDC2	2:18,525,903–18759573	plexin domain containing 2
ENSGALG00000008591	CACNB2	2:19,119,896–19,343,839	calcium voltage-gated channel auxiliary subunit beta 2
ENSGALG00000036128	ZNF407	2:91,466,387–91,808,773	zinc finger protein 407
ENSGALG00000033168	ENSGALG-00000033168	2:91,686,325–91,690,076	
ENSGALG00000015261	NPBWR1	2:110,084,486–110087007	neuropeptides B and W receptor 1
ENSGALG00000025941	RGS20	2:110,314,102–110333425	regulator of G-protein signaling 20
ENSGALG00000015274	TCEA1	2:110,336,672–110362086	transcription elongation factor A1
ENSGALG00000031869	RP1	2:110,575,597–110697739	retinitis pigmentosa 1 (autosomal dominant)
ENSGALG00000035429	XKR4	2:110,760,621–110984213	XK related 4
ENSGALG00000031835	TMEM68	2:111,010,166–111029857	transmembrane protein 68
ENSGALG00000030767	LOC421125	2:111,032,635–111051679	transmembrane protein 68-like
ENSGALG00000015340	TGS1	2:111,084,380–111107118	trimethylguanosine synthase 1
ENSGALG00000042321	LYN	2:111,134,283–111,181,422	LYN proto-oncogene, Src family tyrosine kinase
ENSGALG00000015836	CEP162	3:77,469,083–77515525	centrosomal protein 162
ENSGALG00000008465	SORCS1	6:26,083,271–26356014	sortilin related VPS10 domain containing receptor 1
ENSGALG00000009289	SLC18A2	6:30,092,809–30110773	solute carrier family 18 member A2
ENSGALG00000033195	IGF2BP2	9:4,590,676–4607425	insulin like growth factor 2 mRNA binding protein 2
ENSGALG00000031128	SENP2	9:4,610,405–4623784	SUMO1/sentrin/SMT3 specific peptidase 2
ENSGALG00000054723	AMOTL2	9:4,625,172–4,641,190	angiomotin like 2
ENSGALG00000001690	GABRB2	13:8,067,187–8204642	gamma-aminobutyric acid type A receptor beta2 subunit

**Table 4:** Functional annotation for overlap genes of ROH islands with selective sweeps

Terms	Term Name	Term ID	*P* value	Gene Number
GO:MF	molecular_function	GO:0003674	8.59E-09	49
GO:MF	ion binding	GO:0043167	4.51E-02	16
GO:BP	cellular process	GO:0009987	5.40E-07	45
GO:BP	mast cell degranulation	GO:0043303	2.10E-02	2
GO:BP	regulation of mRNA metabolic process	GO:1,903,311	2.10E-02	4
GO:BP	regulation of actin polymerization or depolymerization	GO:0008064	2.60E-02	3
GO:BP	plasma membrane organization	GO:0007009	2.60E-02	3
GO:BP	immune response-inhibiting cell surface receptor signaling pathway	GO:0002767	3.65E-02	1
GO:BP	7-methylguanosine cap hypermethylation	GO:0036261	3.65E-02	1
GO:BP	post-embryonic hindlimb morphogenesis	GO:0035129	3.65E-02	1
GO:BP	positive regulation of GTPase activity	GO:0043547	3.65E-02	2
GO:BP	pyridoxal phosphate catabolic process	GO:0032361	3.65E-02	1
GO:BP	positive regulation of dendritic cell apoptotic process	GO:2,000,670	3.65E-02	1
GO:BP	neuromuscular junction development	GO:0007528	3.74E-02	2
GO:BP	striated muscle cell development	GO:0055002	4.24E-02	2
GO:BP	establishment of protein localization to membrane	GO:0090150	4.24E-02	3
GO:BP	regulation of alternative mRNA splicing, via spliceosome	GO:0000381	4.24E-02	2
GO:BP	monoamine transport	GO:0015844	4.24E-02	2
GO:BP	locomotory behavior	GO:0007626	4.24E-02	3
GO:BP	lymphocyte homeostasis	GO:0002260	4.24E-02	2
GO:BP	threonine catabolic process	GO:0006567	4.74E-02	1
GO:BP	negative regulation of mast cell proliferation	GO:0070667	4.74E-02	1
GO:BP	slow endocytic recycling	GO:0032458	4.74E-02	1
GO:BP	actin rod assembly	GO:0031247	4.74E-02	1
GO:BP	inhibitory chemical synaptic transmission	GO:0098977	4.74E-02	1
GO:BP	membrane depolarization during atrial cardiac muscle cell action potential	GO:0098912	4.74E-02	1
GO:BP	inner ear receptor cell development	GO:0060119	4.74E-02	2
GO:BP	negative regulation of toll-like receptor 2 signaling pathway	GO:0034136	4.74E-02	1
GO:BP	membrane depolarization during AV node cell action potential	GO:0086045	4.74E-02	1
GO:BP	cardiac muscle thin filament assembly	GO:0071691	4.74E-02	1
GO:BP	protein localization to cilium	GO:0061512	5.00E-02	2
GO:CC	cellular_component	GO:0005575	1.81E-06	45
GO:CC	galectin complex	GO:1,990,724	2.21E-02	1
GO:CC	RNA polymerase II, holoenzyme	GO:0016591	3.84E-02	2

### Combination of ROH and selection signature analysis with GWAS

**Fig. 4:**
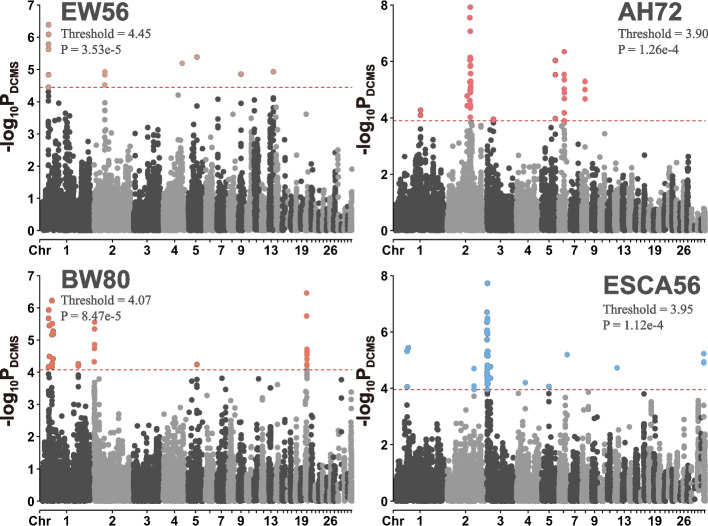
Manhattan plots of selective sweeps for EW56, BW80, AH72 and ESCA56. Selective sweeps detected by DCMS on whole genome for EW56, BW80, AH72 and ESCA56. Abbreviations: AH, albumen height; BW, body weight; ESC: eggshell color; EW, egg weight. The number following the trait indicates the age of week

To investigate the relationship between potentially selected regions and phenotypes, we performed GWAS and two-tailed analyses for 44 phenotypes on this population. We compared differences of the incidence of SNP in ROH segments and detected selection signatures with DCMS methods between the highest and lowest 10% subpopulations of samples for each phenotype, and combined these results with GWAS. First, we found that for all phenotypes, the average differences of the frequency of SNP appearing in ROH between the high and low subpopulations were almost zero, indicating that there were no obvious differences in genome homozygosity between two subpopulations (see Additional File 2, Table S2, where positive values indicate a higher frequency of SNPs occurring in the ROH segments of the low group, with the number representing the frequency difference; negative values indicate a higher frequency of SNPs in ROH segments in the high group). Then, we scanned the genome using FST, Pi ratio (Pi ratio = Pi(low) / Pi(high)), and cross-population extended haplotype homozygosity (XP-EHH, high group was set to be reference subpopulation) to analyze the genomic differentiation between two subpopulations for each phenotype, respectively, and established a DCMS framework individually to obtain *P*-values for each genomic window (Fig. 4). We retained genomic windows with FDR-corrected *P* values of less than 0.05 as potential selection signatures for each  phenotype. In addition, GWAS was performed to identify the significant SNPs for each phenotype on the whole population. In summary, we identified 1,682 significant and 4,095 suggestive significant SNPs (see Additional file 3 Table S3 and Additional file 4 Figure S1). Following GWAS, we also defined 29 QTL regions under 1 Mb in length, within 20 phenotype models (Table 5).

**Table 5:** QTL regions and lead SNPs of phenotypes

Trait	Lead SNP	Position	*P* value	QTL left	QTL right
AH72	rs736230645	5:55,265,697	7.42E-11	55,001,211	55,289,370
BW56	1:71,807,817	1:71,807,817	4.37E-07	71,398,809	71,982,505
BW80	rs316220739	9:15,536,731	2.27E-07	15,408,642	15,853,453
BW80	20:7,110,620	20:7,110,620	2.47E-07	6,610,644	7,610,553
BWAFE	7:226,974	7:226,974	3.03E-08	36,935	835,038
ESCA36	rs313161340	6:32,610,280	6.28E-08	32,571,017	32,749,221
ESCA36	8:2,090,790	8:2,090,790	1.80E-07	1,590,889	2,590,685
ESCA56	3:2,988,169	3:2,988,169	2.62E-08	2,563,304	3,983,803
ESCI36	rs739533938	6:32,668,898	1.86E-07	32,482,856	32,759,520
ESCI56	3:2,988,169	3:2,988,169	1.70E-07	2,488,286	3,484,831
ESCI56	8:6,305,498	8:6,305,498	1.95E-07	5,805,555	6,805,492
ESCI80	3:2,988,169	3:2,988,169	5.09E-07	2,488,286	3,484,831
ESCI80	8:6,312,140	8:6,312,140	2.77E-07	5,812,371	6,812,090
ESCL36	rs731984255	8:1,872,357	1.99E-07	1,372,448	2,372,313
ESCL56	3:2,988,169	3:2,988,169	6.48E-08	2,488,286	3,484,831
ESCL56	8:6,305,498	8:6,305,498	1.03E-07	6,265,397	6,317,241
ESCL72	8:6,293,110	8:6,293,110	1.16E-07	6,201,681	6,432,209
ESCL80	3:2,988,169	3:2,988,169	5.98E-08	2,488,286	3,484,831
ESCL80	8:6,312,140	8:6,312,140	8.70E-09	5,812,371	6,812,090
ESS36	rs734838923	3:103,651,372	3.22E-07	103,151,587	104,151,356
ESS36	rs735838278	8:6,303,273	3.33E-07	5,803,530	6,803,257
EW36	rs733232315	5:57,609,532	1.50E-07	57,110,085	58,109,515
EW56	rs732195048	1:25,343,938	1.44E-08	24,902,556	25,908,677
EW56	6:5,397,806	6:5,397,806	5.83E-07	4,897,817	5,897,731
EW72	1:25,436,089	1:25,436,089	4.08E-11	25,143,248	25,907,962
EW80	1:25,705,664	1:25,705,664	9.61E-08	25,205,864	26,205,603
EW80	24:5,133,917	24:5,133,917	2.72E-07	4,645,990	5,633,270
SINS36	2:63,008,967	2:63,008,967	5.37E-07	62,508,989	63,508,907
SINS56	rs316665180	23:2,835,996	4.50E-08	2,809,164	3,332,578

The number following the trait indicates the age of week

*Abbreviations*: *AH* Albumen height (mm), *BW* Body weight (g), *AFE* Age at first egg, *ESC* Eggshell color, *ESS* Eggshell strength, *EW* Egg weight (g), *SINS* Eggshell gloss

**Fig. 5:**
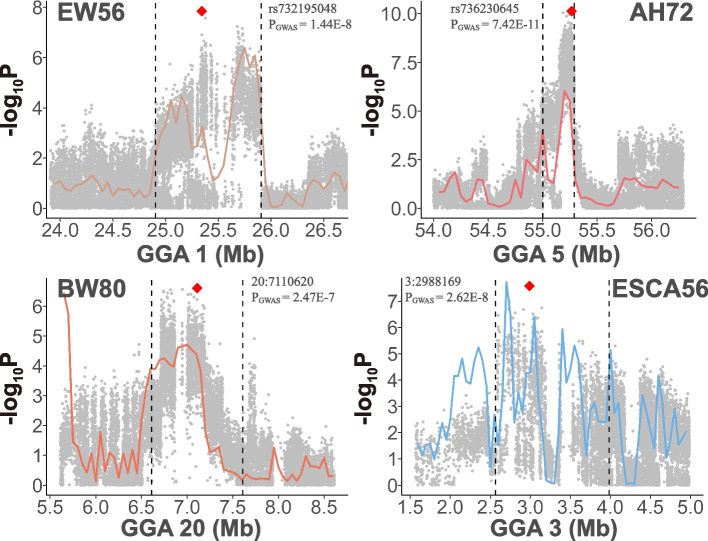
Regional point and line plots of GWAS and selective sweeps. The combination of the regional point plots of GWAS with the line plots of selective sweeps for EW56, BW80, AH72 and ESCA56. Within the black vertical dashed line are the QTL regions defined through GWAS results. Red diamond dots indicate lead SNP within the corresponding QTL regions. The colored lines in each subfigure represent the *P*-values of the windows calculated by DCMS methods. Abbreviations: AH, albumen height; BW, body weight; ESC: eggshell color; EW, egg weight. The number following the trait indicates the age of week

**Fig. 6:**
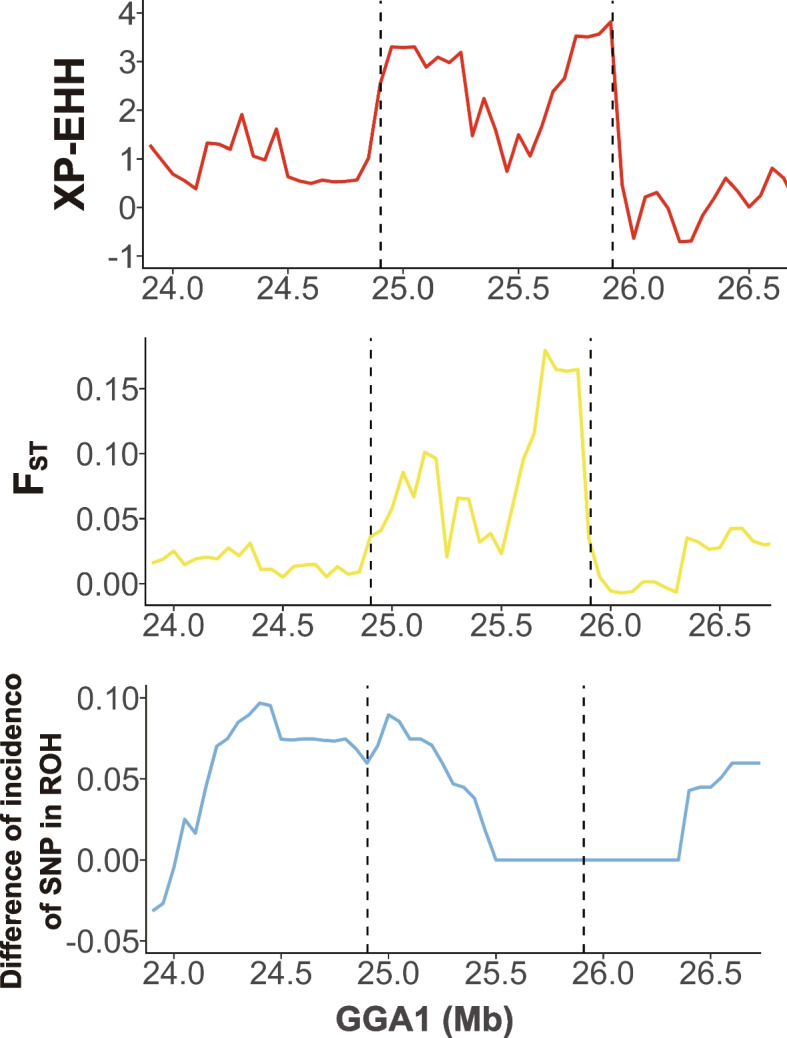
Line plots of multi selective sweeps methods on the QTL associated with EW56. The upper, middle, and lower line plots represent the trends of the XP-EHH, FST and the differences of incidence of SNP in ROH segments near the QTL region associated with EW56, respectively

Among these 29 QTL regions, we found that 13 overlapped with selection signatures identified by the DCMS method, involving multiple time points for EW, ESC and SINS, as well as BW80, AH72 and ESS36 (Fig. 5). Taking egg weight as an example (Fig. 6), we located the QTL near 25 Mb on GGA1. This region was highlighted not only by the DCMS methods but also exhibited selection signatures through the XP-EHH and FST methods. Since the standardized XP-EHH value is greater than 2, it suggests a higher extended haplotype homozygosity in this region of the low egg weight subpopulation. Furthermore, in this region, it was also observed that SNPs within ROH segments occur at a higher frequency in the low egg weight subgroup than the high (4.4 ~ 8.9%), implying that the low egg weight subpopulation has a higher degree of homozygosity in this region. In addition, six genes are located within this QTL region: CAPZA2, MET, CAV1, CAV2, TES, TFEC, and we performed functional annotation for them (Table 6).

**Table 6:** Functional annotation for genes located in QTL regions associated with egg weight

Terms	Term Name	Term ID	*P* value	Gene Number
GO:MF	potassium channel inhibitor activity	GO:0019870	1.18E-02	1
GO:MF	protein dimerization activity	GO:0046983	1.18E-02	3
GO:MF	protein tyrosine kinase inhibitor activity	GO:0030292	1.63E-02	1
GO:MF	peptidase activator activity	GO:0016504	2.46E-02	1
GO:MF	transmembrane receptor protein tyrosine kinase activity	GO:0004714	3.67E-02	1
GO:MF	protein phosphatase binding	GO:0019903	4.35E-02	1
GO:BP	plasma membrane raft assembly	GO:0044854	9.23E-05	2
GO:BP	negative regulation of cellular process	GO:0048523	1.25E-04	6
GO:BP	intracellular nitric oxide homeostasis	GO:0033484	4.53E-03	1
GO:BP	negative regulation of anoikis	GO:2,000,811	1.26E-02	1
GO:BP	muscle cell cellular homeostasis	GO:0046716	1.51E-02	1
GO:BP	positive chemotaxis	GO:0050918	1.65E-02	1
GO:BP	regulation of cytosolic calcium ion concentration	GO:0051480	1.72E-02	1
GO:BP	membrane depolarization	GO:0051899	1.99E-02	1
GO:BP	lipid storage	GO:0019915	2.28E-02	1
GO:BP	triglyceride metabolic process	GO:0006641	2.40E-02	1
GO:BP	cholesterol homeostasis	GO:0042632	2.47E-02	1
GO:BP	response to calcium ion	GO:0051592	2.86E-02	1
GO:CC	caveolar macromolecular signaling complex	GO:0002095	2.42E-06	2
GO:CC	focal adhesion	GO:0005925	2.72E-05	3
GO:CC	cell cortex	GO:0005938	2.12E-03	2
GO:CC	basal plasma membrane	GO:0009925	4.25E-02	1

## Discussion

In the current study, we utilized WGS data for ROH, selective sweeps and GWAS analyses in a population of 686 Rhode Island Red hens. With this study, we aim to explore the impact artificial selection might have on the genomes of layers and provide a theoretical foundation for specific breeding practices.

It is important to emphasize that this population has a relatively small number of founders and has undergone several generations of intense confinement selection for egg-related traits. These are important contextual factors for interpreting and discussing our results.

Compared to the results of previous studies on chicken [15, 17–19], the distribution and number of ROH in this population did not exhibit significant differences. ROH segments are commonly found on larger chromosomes, and most are classified as short in length (< 3 Mb). It is generally believed that longer ROH indicate inbreeding or a strong selection pressure, while shorter ROH can reflect the population structure of ancestors [7, 8]. In addition, we have observed a relatively higher proportion of long ROH (> 3 Mb) on smaller chromosomes; however, there is no clear explanation for this phenomenon yet.

One common application of ROH analysis is to assess inbreeding levels within a population. In our population, the value of F(ROH) was much larger than the other methods, which is relatively rare in various previous animal studies [3, 13, 20]. Comparative analysis of various methods revealed that F(ROH) and F(PED) exhibit relatively low correlations with alternative approaches, whereas F(GRM) displays a higher correlation with F(HOM). The discrepancy may arise from the use of different SNP datasets in the computation process—F(GRM) and F(HOM) typically employ loci independently filtered for LD [16]. In fact, when we used independent SNPs for detection of ROH, we found that the value of F(ROH) decreased, while the correlation with F(HOM) and F(GRM) increased. In the commercial breeding, pedigrees are conventionally employed to circumvent inbreeding, culminating in diminished inbreeding coefficients. Nonetheless, genomic analysis indicates that sustained intensive selective breeding across multiple generations can escalate genomic homozygosity, thereby exacerbating inbreeding levels. This phenomenon potentially accounts for the pronounced disparity in F(PED) relative to other genomic-based inbreeding coefficients. Further, our research indicates that individuals with more closely related parents possess an increased number of ROH segments and elevated F(ROH) values, confirming that ROH can measure the degree of inbreeding.

In analyzing the entire population, our objective was to pinpoint regions and corresponding genes under strong selection pressure by investigating genomic regions featuring overlaps between ROH islands and selective sweeps. We commenced by determining the frequency of each SNP within ROH segments, isolating the top 1% of SNPs genome-wide to identify 23 ROH islands. The findings revealed that both the SNP density and the MAF on these islands were considerably lower than the overall genomic mean. We speculate that reduced SNP density may aid ROH detection, and a diminished MAF indicates a propensity for SNP fixation within ROH islands.

DCMS strategy effectively amalgamates diverse selection signatures methods within a population into a single score, enhancing the precision of signal detection [21]. Within this framework, the methods Pi, Tajima’s D, and iHS were employed. We designated the top 1% of windows by DCMS *P*-value as potential signals of selection. Our analysis revealed that over half of ROH islands coincide with these candidate regions, culminating in the delineation of 60 genes. Subsequent functional annotation indicated that these pathways could influence a range of physiological activities in layers, encompassing growth, immunity, disease resistance, cardiovascular and neurological functions, kinesthetic capabilities, behavior, and metabolic processes at the cellular level. We have discovered several genes that may be related to the physiological activities of laying hens. The gene LARGE1 (Like-Glycosyltransferase 1) is associated with pathways related to ion channels and signal transduction in the body of laying hens. This gene encodes a glycosyltransferase that participates in the modification of glycoproteins. It has been reported as a gene potentially related to the abundance of intestinal microbiota in chickens, influencing the deposition of abdominal fat in chickens [22]. LYN (LYN Proto-Oncogene, Src Family Tyrosine Kinase) is a tyrosine kinase involved in various pathways related to immunity and cell apoptosis, and is also associated with growth [23] and aging [24]. These results describe the changes that occur in genome on whole population when it is subjected to artificial selection, yet it is difficult to directly relate to a specific production trait or breeding purpose.

The above findings mainly focused on regions of the genome that have been subjected to selection in the entire population, but did not address specific traits. To gain a deeper understanding of the impact of artificial selection on specific traits and associated genes, we conducted a two-tailed analysis. The analysis included: comparing the ROH islands and computing multiple selection signatures and building the DCMS framework between high and low subgroups, and combining these results with GWAS on each phenotype. We first found that in the high and low subgroups for most traits, the mean of the frequency differences of SNPs in ROH was close to zero, with extremes ranging from 0.22 to 0.38. These results suggest that there is no difference in the genomic homozygosity between high and low subgroups at the whole-genome level, though significant variations may exist in localized regions.

GWAS is capable of identifying common variants that explain genetic variation, and through it, we identified thousands of SNPs associated with different traits and defined QTL regions. We then explored the selection signatures by DCMS between high and low subgroups of each phenotype and corrected the results using FDR. Our aim was to find selection signatures in regions significantly associated with traits (QTL). Overall, we defined 29 QTL regions in 20 phenotypes (related to 8 traits, including AH, BW, ESCA, ESCI, ESCL, ESS, EW and SINS), and 13 QTLs overlapped with the significant regions in DCMS, which meant that these regions significantly associated with the traits were at the same time selection signatures identified by DCMS. In addition, since XP-EHH could be used to determine which subgroups had higher extended haplotype homozygosity, while ROH islands analysis could determine which had higher homozygosity, it might be possible to link the targets of artificial selection to the actual changes on the genome. Using EW56 as an example, we found significant DCMS results near the QTL region associated with EW56, implying that there might be genomic differences between the high and low EW56 subpopulations. The XP-EHH results were greater than 2, this suggested that the extended haplotype homozygosity was higher in the low EW56 subpopulation. In addition, the ROH island analysis also indicated that the degree of homozygosity of low EW56 subpopulation was higher near this region. Since the aim of artificial selection in this population is to reduce egg weight, our results seem to match this aim, but the exact relationship remains unknown.

Six genes are located within the QTL regions, and we have performed functional annotation on them. CAPZA2 and TES are involved in cell adhesion and regulating the cytoskeleton, affecting cell shape and movement. F-actin-capping protein subunit alpha-2 (CAPZA2) is a cytoskeleton assembly-associated protein that may be associated with the formation of small intestinal microvilli in poultry [25, 26]; Testin (TES) testosterone is a protein that is expressed in virtually all normal human tissues, and it plays an important role in its cell motility, adhesion and cytoskeleton [27]. Caveolin-1 (CAV1) is involved in several physiological activities such as signaling, apoptosis, and lipid metabolism. It has been reported to be associated with eggshell quality and organismal aging [28]. Caveolin-2 (CAV2) and CAV1, are members of the CAV family and are involved in the formation of lipid rafts (cholesterol-rich microdomains within membranes). They play a role in a variety of signaling, lipid metabolism, and cell protection from programmed death [29]. TFEC, as a transcription factor, may regulate genes related to cell differentiation and metabolism and has been reported to be expressed in chicken macrophages [30]. These genes are associated with the biological processes listed in Table 6, which may indirectly or directly influence the egg weight of laying hens. This includes (a) the modulation of membrane channels and signal transduction, such as potassium channel inhibition and calcium concentration regulation, which may affect muscle contraction and the function of the oviduct; (b) regulation of cell survival and programmed cell death, which may impact on follicle survival and lipid metabolism, related to the size and quality of the egg; © regulation of the cytoskeleton and adhesive structures, affecting cell morphology, differentiation, and the physical stability of follicles; (d) lipid and energy metabolism, such as lipid storage and cholesterol balance, which may play an important role in regulating the composition and size of the yolk.

## Conclusions

In this research, we used WGS data to perform a genomic analysis of Rhode Island Red inbred lines of laying hens, employing ROH and selection signatures analyses. We detected 60 candidate genes within the intersection of ROH and selection signatures, potentially influencing productive attributes related to growth, immune response, disease resistance, cardiovascular health, and neurological functions in laying hens. Integrating both two-tailed analyses with GWAS identified multiple QTL subjected to selection for various phenotypes. Specifically, for egg weight, a QTL was pinpointed near the 25MB region on GGA 1. And both XP-EHH and ROH analyses indicated higher degree of extended haplotype homozygosity and genomic homozygosity in the low egg weight subpopulation near this region, consistent with the direction of artificial selection. Functional annotation of six genes (CAPZA2, MET, CAV1, CAV2, TES, TFEC) within this QTL indicated associations with vital physiological pathways, such as signal transduction, apoptotic processes, and lipid metabolism. Overall, our findings enhance the comprehension of the genome of layers and inform for poultry breeding programs.

## Methods

### Samples and whole genome sequencing

The population used in this study was obtained from a Rhode Island Red pure line in a commercial laying breeding program in Beijing, China. This line had been subjected to fifteen consecutive generations of intensive selection with selection indexes including EN, ESC and egg quality traits. The breeding stock was selected and reproduced for one generation per year, with 80 ~ 100 sire families per generation. Inbreeding was avoided in mating plans based on the pedigree. A total of 686 female chickens with full pedigree records over the past six generations and complete measurements of body weight and egg-related trait at different ages were used in this study. For each chicken, DNA was extracted from a volume of approximately 2 ml venous blood, which was collected from the wing and then placed in an anticoagulation tube with EDTA. The integrity of the DNA was verified, and whole-genome sequencing was performed using an Illumina HiSeq 2500 Sequencer (Illumina, Inc., San Diego, CA, USA). Paired-end reads of 150 bp were generated for each sample.

### Data processing and quality control

Raw sequencing data were processed to acquire high-quality single nucleotide polymorphisms (SNPs) by adhering to the following protocols. Low-quality reads were filtered using FastQC v0.11.9 software [31]. Clean reads were then aligned to the *Gallus gallus* 6.0 reference genome using the Burrows‒Wheeler Alignment (BWA) v0.7.17 tool with default settings [32]. Potential duplicate reads were removed using the Picard toolkit, and the resulting alignments were indexed via SAMtools v1.17 [33]. Genome Analysis Toolkit (GATK) v4.2.3 was employed to process the alignments according to best practices [34]. To derive high-quality SNPs, a minimum quality score of 20 was applied to both bases and mapped reads for variant calling. SNPs from each bird were combined, and the resulting data were filtered using the GATK Variant Filtration module by applying stringent criteria: quality by depth > 5.0, mapping quality score > 40.0, FS < 60.0, MQRankSum > -12.5, ReadPosRankSum > -8.0, and excluding any three SNPs clustered within a 10 bp window. Subsequently, we employed PLINK v1.9 [35] to filter the SNP data using set parameters: a sample call rate exceeding 0.9, a SNP call rate over 0.9, and MAF greater than 0.01. Following filtration, the remaining SNPs and individuals were earmarked for imputation via BEAGLE v5.2 [36]. We then reperformed the PLINK v1.9 analysis, adhering to the same criteria previously mentioned. After these procedures, a comprehensive total of 5,904,820 SNPs spread across 32 chromosomes from 686 birds remained for subsequent analysis. Finally, these phased and filtered SNP data were annotated with SNPEff v5.2 [37] utilizing the chicken reference genome.

### Detection of runs of homozygosity (ROH) and calculation of the inbreeding coefficient

Two studies were referenced for ROH setting [12, 38], and PLINK v1.9 was used for ROH detection with the following parameters: a minimum length of 500 Kb in an ROH (-homozyg-kb 500), a minimum of 50 SNPs in an ROH (-homozyg-snp 50), the minimum SNP density set to 1 SNP per 50 kb (-homozyg-density 50), and the maximum gap between two consecutive SNPs set to 1000 kb (-homozyg-gap 1000). For a sliding window of 50 SNPs, an allowance of no more than 5 missing SNPs per window (-homozyg-window-missing 5) and a maximum of one heterozygous SNP per window (-homozyg-window-het 1) were set.

Following ROH detection, we computed the coefficient of inbreeding using four different methods: (a) F(ROH), calculated as the sum of ROH segment lengths divided by the whole genome length; (b) F(PED), computed based on the pedigree using CFC software [39]; (c) F(GRM), the result of the diagonal of the genomic relationship matrix (GRM) constructed by GCTA v1.26.0 [40]; and (d) F(HOM), based on the homozygous sites by PLINK v1.9. We compared the results of all four methods in R (https://www.R-project.org/) to evaluate the degree of inbreeding and calculated the correlation coefficients of F(ROH) with F(PED), F(GRM), and F(HOM).

### Detection of ROH islands

To delineate the genomic regions exhibiting the strongest association with ROH, we quantified the prevalence of SNPs within ROH by enumerating the occurrences of a specific SNP in an ROH across a diverse set of individuals. Subsequently, we selected the top 1% of SNPs demonstrating a prevalence exceeding 67.93% and combined proximate SNPs into genomic regions that were representative of ROH islands, which were then subjected to in-depth investigations. This result is presented by a Manhattan plot. All Manhattan plots in this paper were generated by the R packages CMplot [41] and ggplot2 [42].

### Selective sweep identification and De-correlated composite of multiple signals

In addition to the analysis of selection signatures for within-population, we designed a two-tailed analysis to discover genomic differences between high- and low-level individuals for each trait. Before the detection for selection signatures, we placed the individuals ranking in the top and bottom 10% for each trait into the high- and low-level subgroups of this trait. To detect selection signatures within our population or between subgroups of each trait, we employed various strategies to examine the entire genome. Using VCFtools v0.1.16 [43], we computed fixation index (FST), nucleotide diversity (Pi) and Tajima’s D values with a sliding window approach, setting the window size to 50Kb and the step size to 25Kb for FST and Pi, with Tajima’s D also analyzed across 50Kb windows. And the Pi ratio is equal to the Pi value for each window in the low-level subgroup divided by the Pi value in the high-level subgroup (Pi ratio = Pi(low) / Pi(high)). Further, we determined genome-wide XP-EHH [44] and iHS values [45] using the –*xpehh* and *-ihs* command in the Selscan v1.3.0 software [46], normalized these values with the -norm command, and obtained the average XP-EHH and iHS value for each 50 Kb region. And in XP-EHH analysis, high subgroup was set to be reference subpopulation.

We incorporated Tajima’s D, Pi and iHS in the intra-population De-correlated composite of multiple signals (DCMS) [21] framework for detecting selection signatures of whole population, and FST, Pi ratio and XP-EHH in the inter-subpopulation DCMS framework for genomic differentiation between high- and low-level subgroups of each trait. In every DCMS, we calculated this statistic for each 50kb window using the MINOTAUR package in R [47]. The analysis steps were performed with reference to other studies [48–50]. To compute genome-wide *P*-values, we employed the *stat_to_pvalue* function, performing a left-tailed test for Pi, Tajima’s D, and iHS (setting two.tailed = FALSE, right.tailed = FALSE) and a right-tailed test for FST, Pi ratio and XP-EHH (setting two.tailed = FALSE, right.tailed = TRUE). An n × n correlation matrix was generated across these statistics utilizing the *covNAMcd* function in the rrcovNA R package (with parameters alpha = 0.75, nsamp = 300,000). This matrix provided the basis for calculating genome-wide DCMS values via the *DCMS* function in the MINOTAUR package. Subsequent to the DCMS value computation, we fitted a robust linear model to these values and normalized the distribution using the *rlm* function (with model dcms ~ 1) from the MASS R package. Finally, we applied the *pnorm* function to calculate *P*-values for the DCMS statistics and identify candidate sweep regions by evaluating the empirical distribution’s top 1%. For the DCMS between high- and low-subgroups, we applied the *p.adjust* function to conduct FDR correlation for the significant intervals associated with traits.

Then, we used BEDTools v2.26.0 [51] *intersect* to identify SNPs that were located in both ROH islands and potentially selected regions with low DCMS *P*-values. These SNPs were regarded as putative selection signatures and annotated to candidate genes and reported QTLs.

### Functional annotation

We employed the online website g:Profiler (https://biit.cs.ut.ee/gprofiler/gost) [52] to retrieve enriched functional terms for these genes, including Gene Ontology (GO) categories and KEGG pathways.

### Genome Wide Association Study (GWAS)

We conducted a genome-wide association study (GWAS) on all 42 traits incorporating all valid samples and SNPs passing the quality control of MAF (0.05) and Hardy–Weinberg test (1e-6), utilizing a univariate linear mixed model (LMM). This analysis was executed with GEMMA (version 0.98.4) software [53]. The statistical model implemented in this investigation is as follows:

$$\mathbf{y}=\mathbf{W}{\varvec{\upalpha}}+\mathbf{x}{\varvec{\upbeta}}+{\varvec{\upmu}}+{\varvec{\upvarepsilon}}$$

Here, **y** represents the phenotypes of 686 individuals; **W** is a matrix of covariates (fixed effects: top five principal components and hatch effects) accounting for population structure, with **α** being a vector of corresponding effects that form the intercept; **x** signifies the marker genotypes and **β** refers to the associated marker effects; **μ** constitutes a vector of random polygenic effects with a covariance structure; and **ε** denotes a vector of random residuals. Additionally, the *P* value from the likelihood ratio test was chosen as a benchmark to evaluate the significance of the association between SNPs and egg traits. The threshold for genome-wide significance was established using a modified Bonferroni correction implemented through the R package simpleM. A total of 84,633 valid SNPs were carried out, and the thresholds of genome-wide significance and suggestive significance were defined as 5.91e-7 (0.05/84,633) and 1.18e-5 (1/84,633), respectively.

### QTL region definition

For each trait, we used GWAS results to define QTL as chromosomal regions where the distance between adjacent pairs of significant variants was less than 1 Mb [54]. Within each locus, we identified the most significant variant as the lead variant. A maximum distance of 0.5 Mb on either side of the lead variant was allowed.

### Supplementary Information

Supplementary Material 1.

Supplementary Material 2.

Supplementary Material 3.

Supplementary Material 4.

## Data Availability

Whole-genome resequencing data are available on the NCBI Sequence Read Archive (SRA) under PRJNA980845.
