# Supplementary material for: Runs of homozygosity and selection signature analyses reveal putative genomic regions for artificial selection in layer breeding
Source: BMC Genomics. 2024 Jun 26;25:638. doi: 10.1186/s12864-024-10551-4 (PMC11210043; doi:10.1186/s12864-024-10551-4)

All Manhattan plots of GWAS for all traits

The threshold of significance = 6.23 and threshold of suggestive significance = 4.93.
Orange points indicate that the SNPs are significant locus for the trait; green points indicate that the SNPs are suggestive significant locus for the trait.

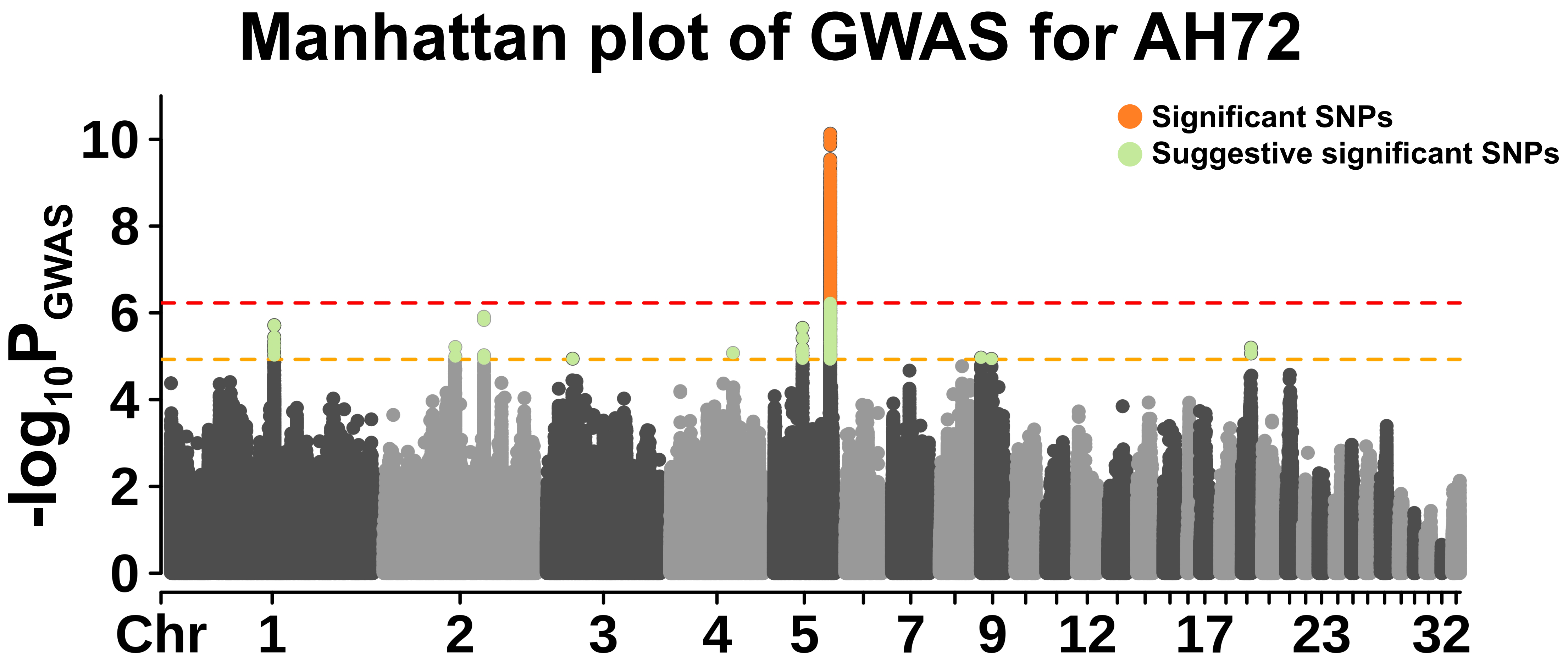

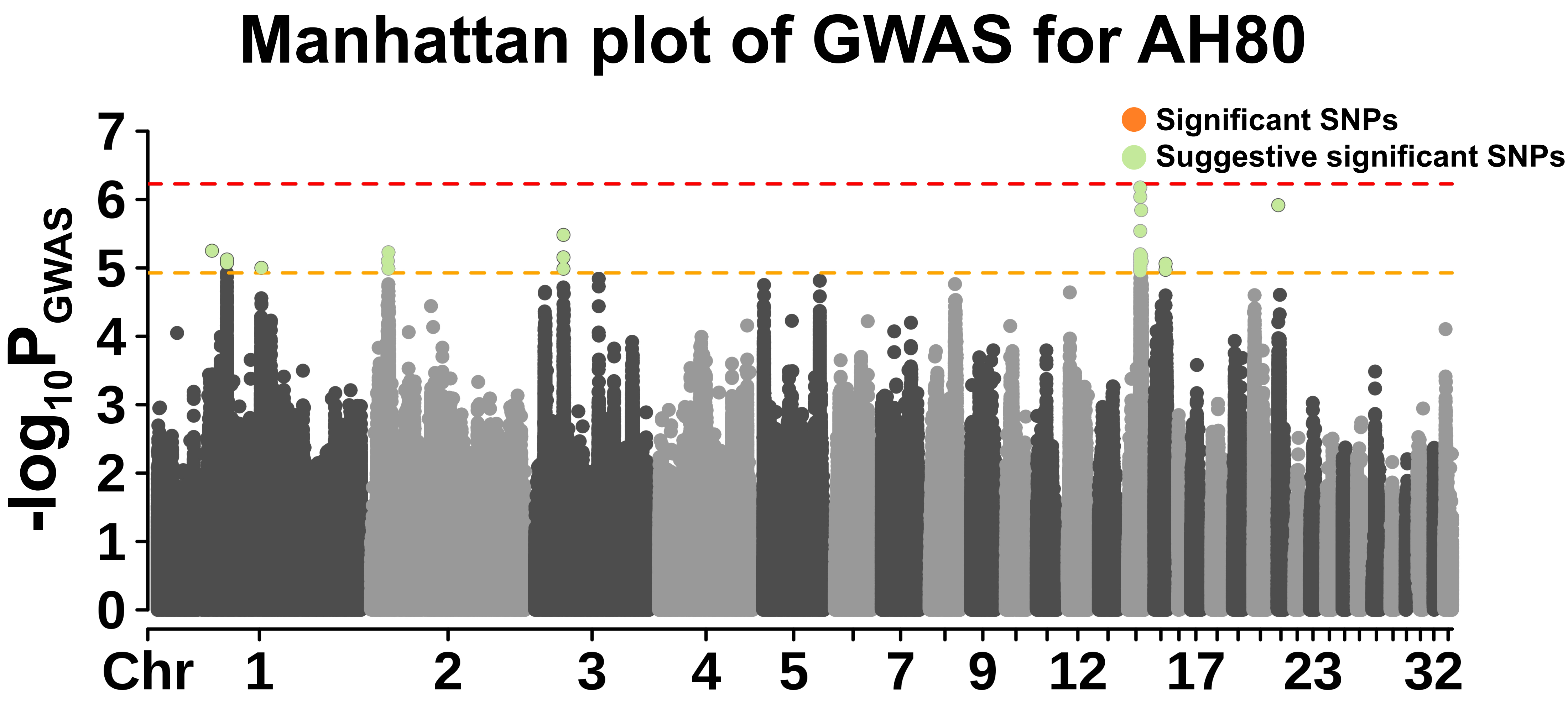

Manhattan plot of GWAS for AH80
Significant SNPs
Suggestive significant SNPs
-log10PGWAS
0
1
2
3
4
5
6
7
Chr
1
2
3
4
5
7
9
12
17
23
32

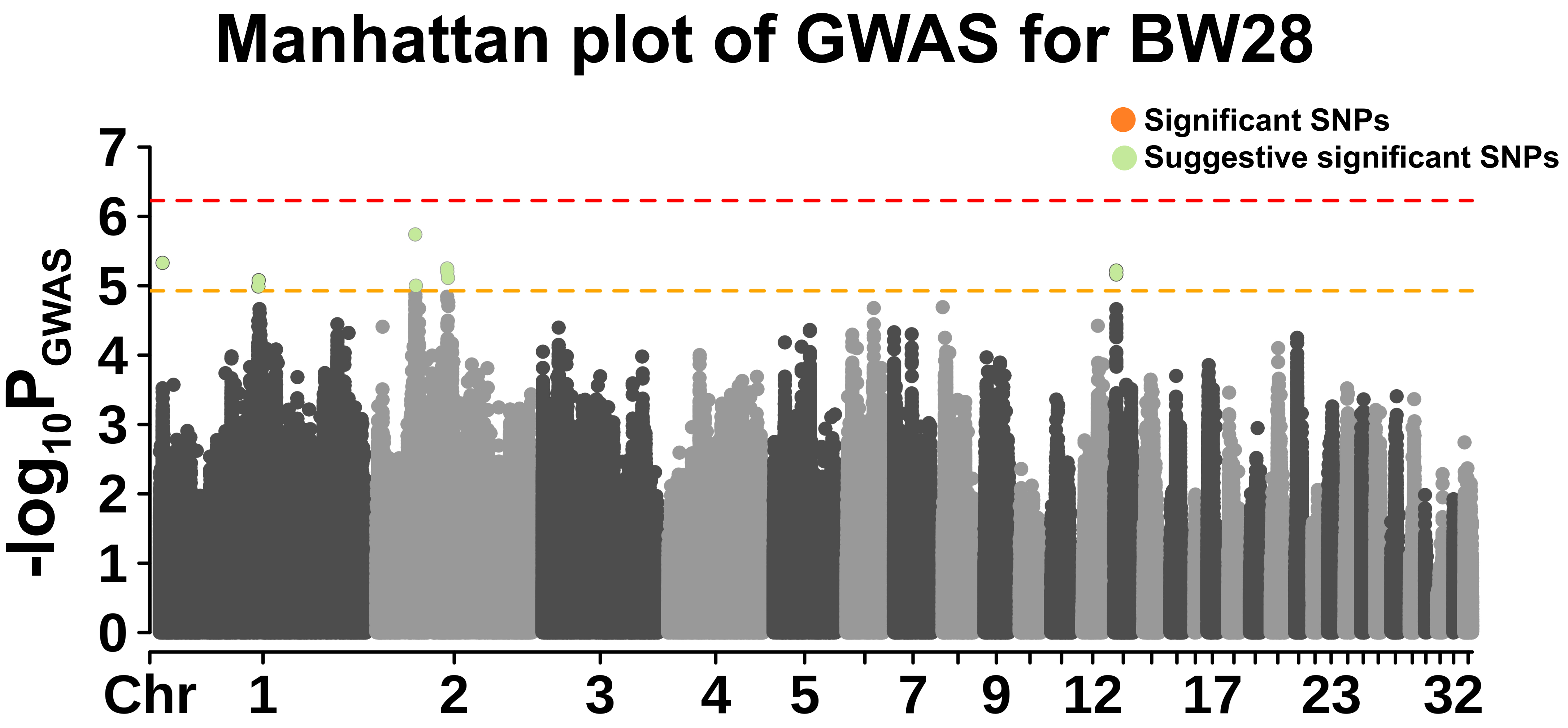

Manhattan plot of GWAS for BW28
Significant SNPs
Suggestive significant SNPs
-log10PGWAS
7
6
5
4
3
2
1
0
Chr
1
2
3
4
5
7
9
12
17
23
32

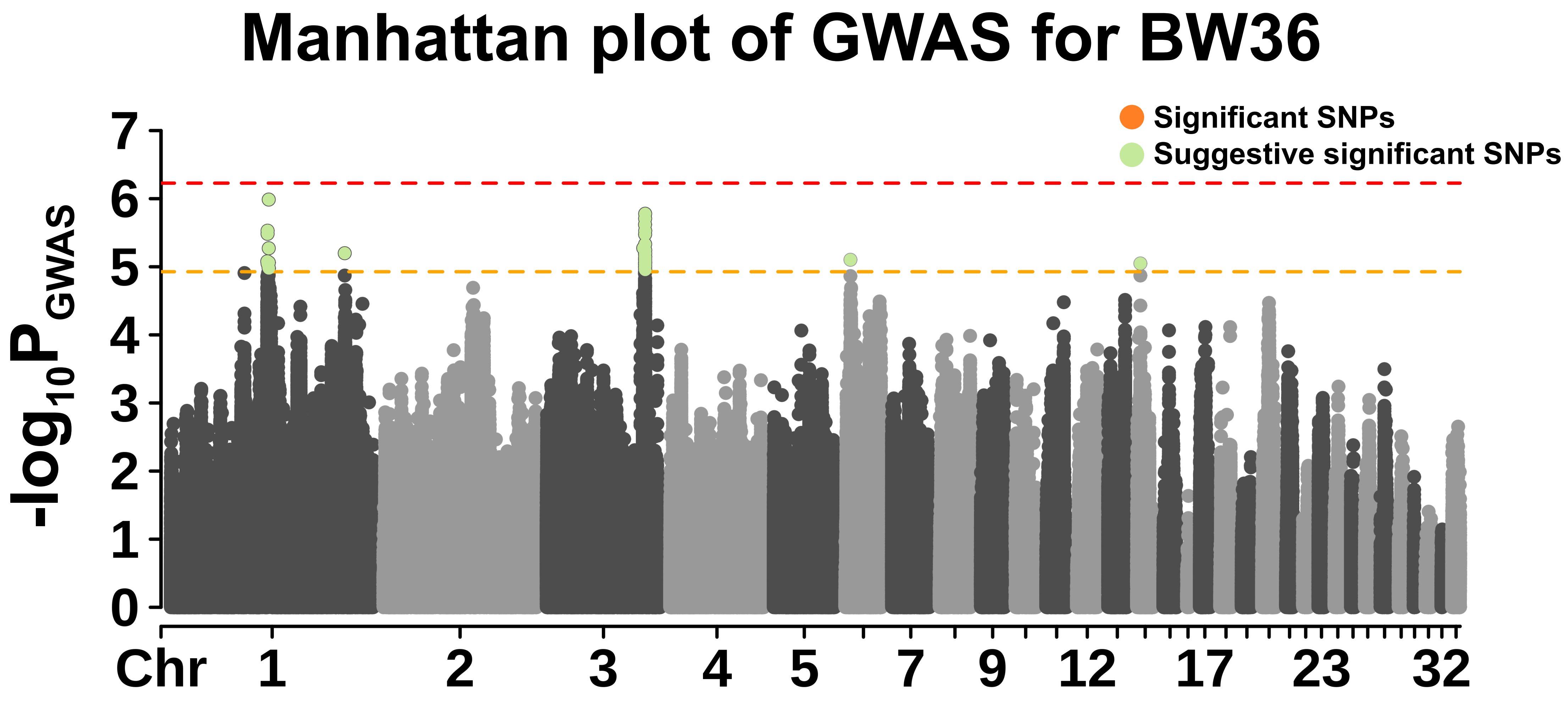

Manhattan plot of GWAS for BW36
Significant SNPs
Suggestive significant SNPs
$-log_{10}P_{GWAS}$
7
6
5
4
3
2
1
0
Chr
1
2
3
4
5
7
9
12
17
23
32

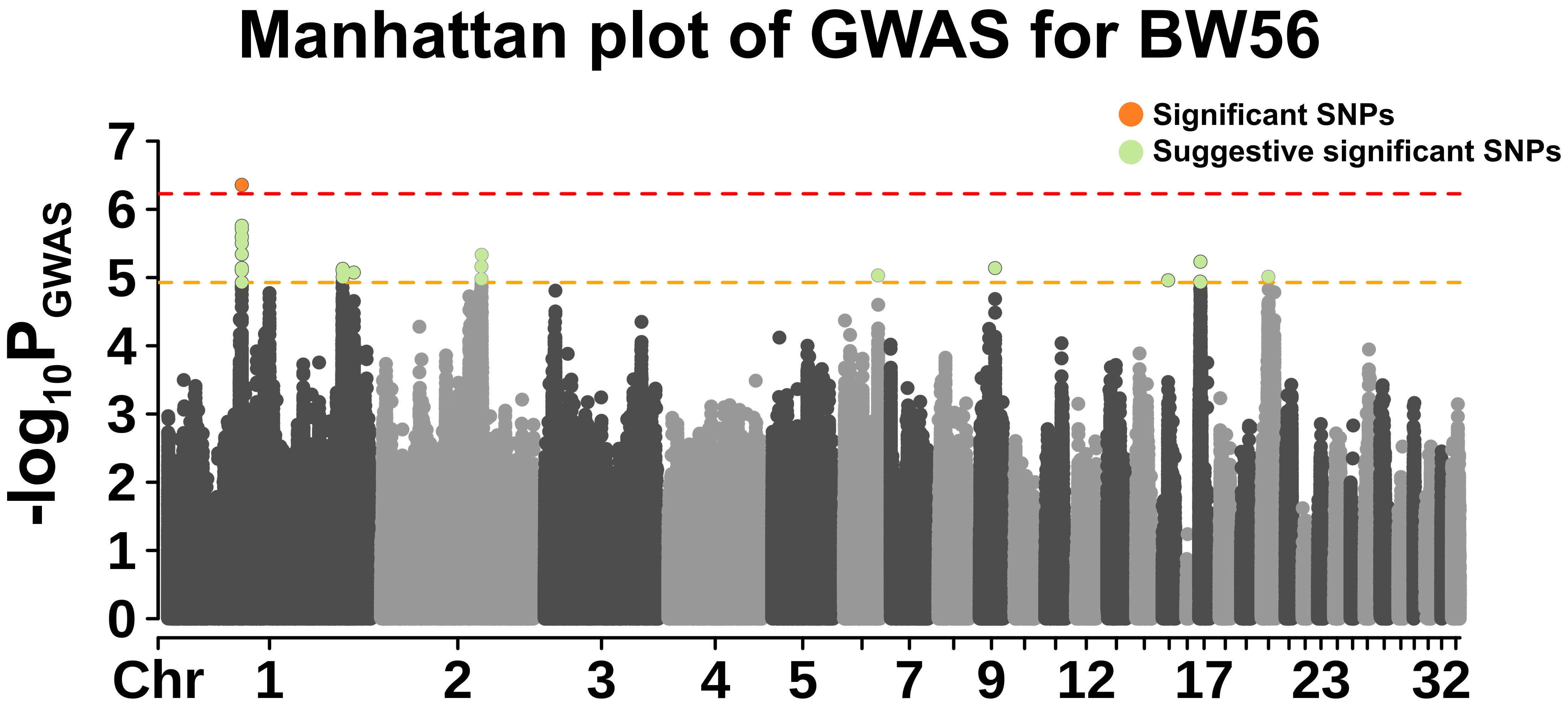

Manhattan plot of GWAS for BW56
Significant SNPs
Suggestive significant SNPs
-log10PGWAS
0
1
2
3
4
5
6
7
Chr
1
2
3
4
5
7
9
12
17
23
32

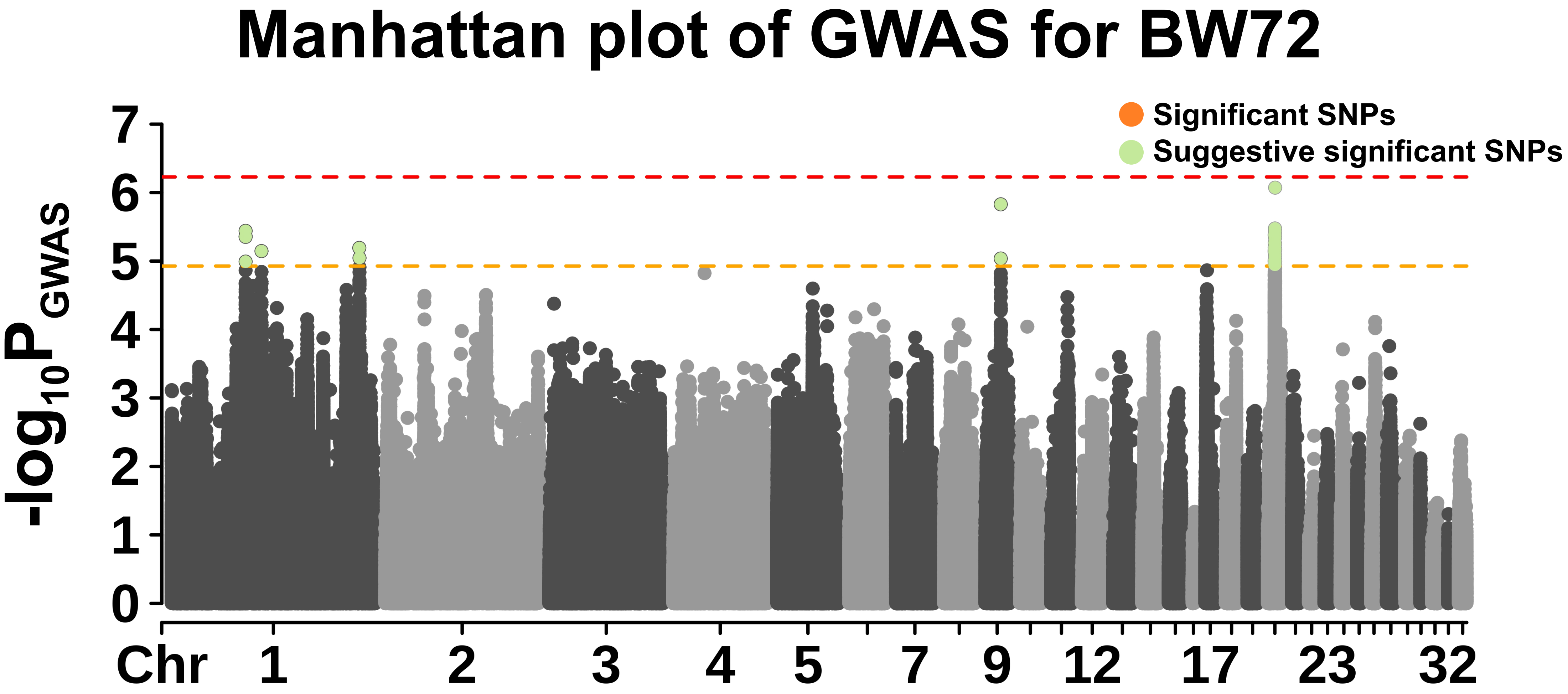
Manhattan plot of GWAS for BW72
Significant SNPs
Suggestive significant SNPs
$-\log_{10}P_{GWAS}$
0
1
2
3
4
5
6
7
Chr
1
2
3
4
5
7
9
12
17
23
32

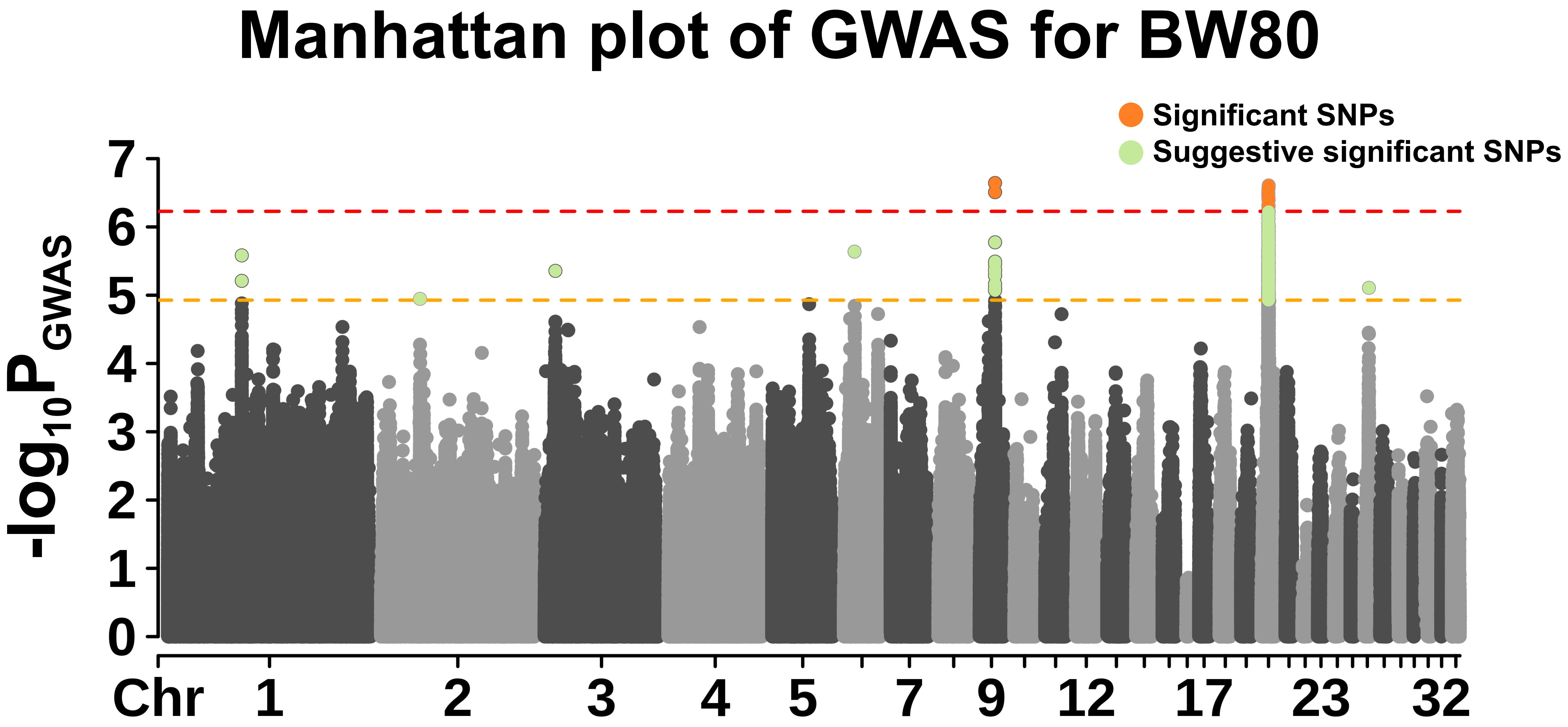

Manhattan plot of GWAS for BW80
Significant SNPs
Suggestive significant SNPs
$-\log_{10}P_{GWAS}$
0
1
2
3
4
5
6
7
Chr
1
2
3
4
5
7
9
12
17
23
32

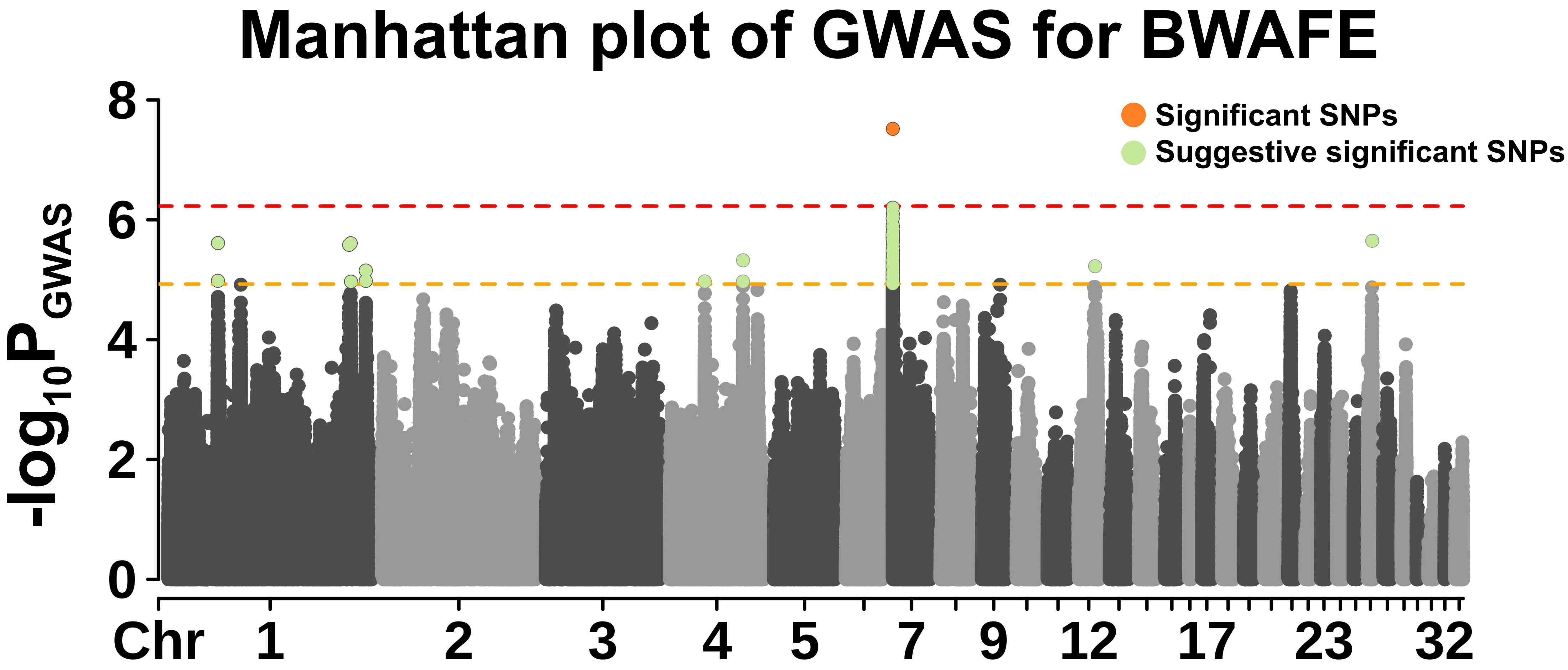

Manhattan plot of GWAS for BWAFE
Significant SNPs
Suggestive significant SNPs
$-\log_{10}P_{GWAS}$
8
6
4
2
0
Chr
1
2
3
4
5
7
9
12
17
23
32

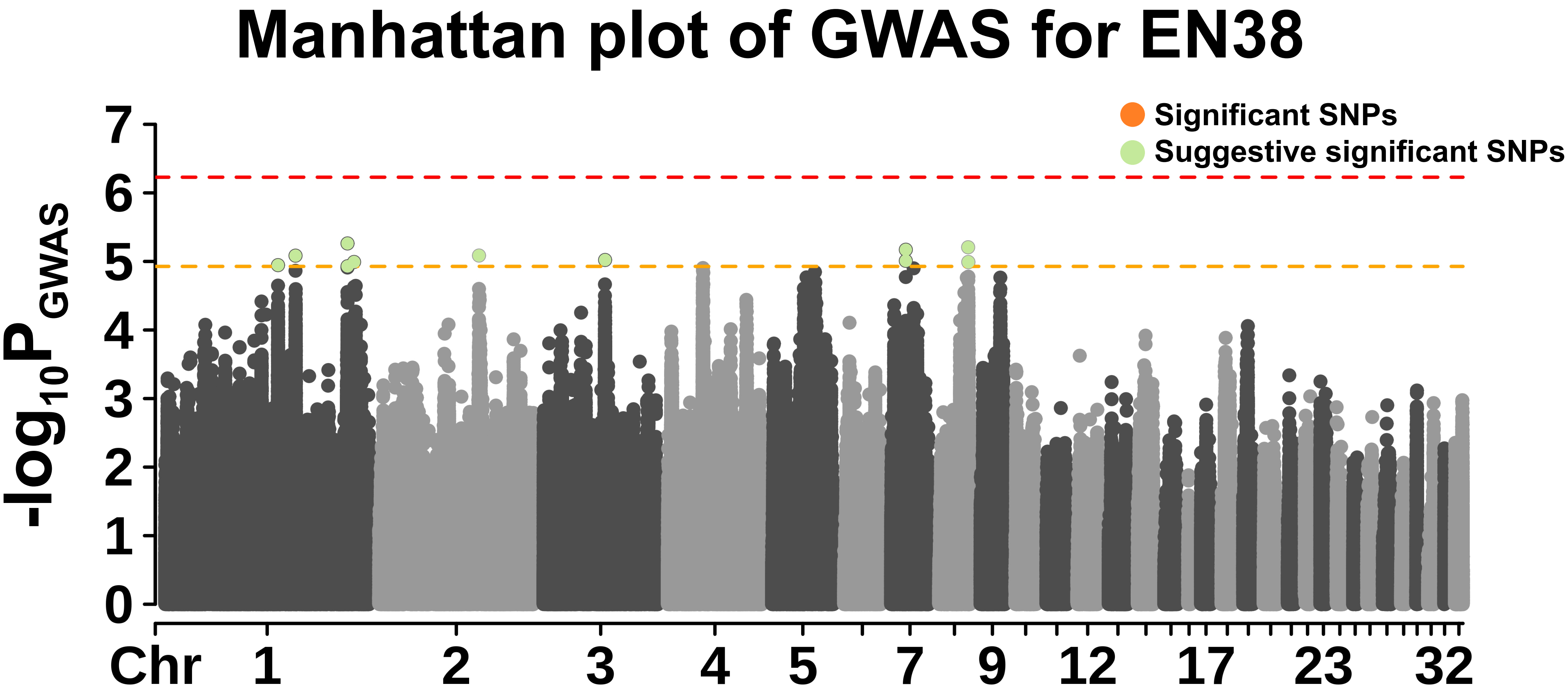

Manhattan plot of GWAS for EN38
Significant SNPs
Suggestive significant SNPs
-log10PGWAS
0
1
2
3
4
5
6
7
Chr
1
2
3
4
5
7
9
12
17
23
32

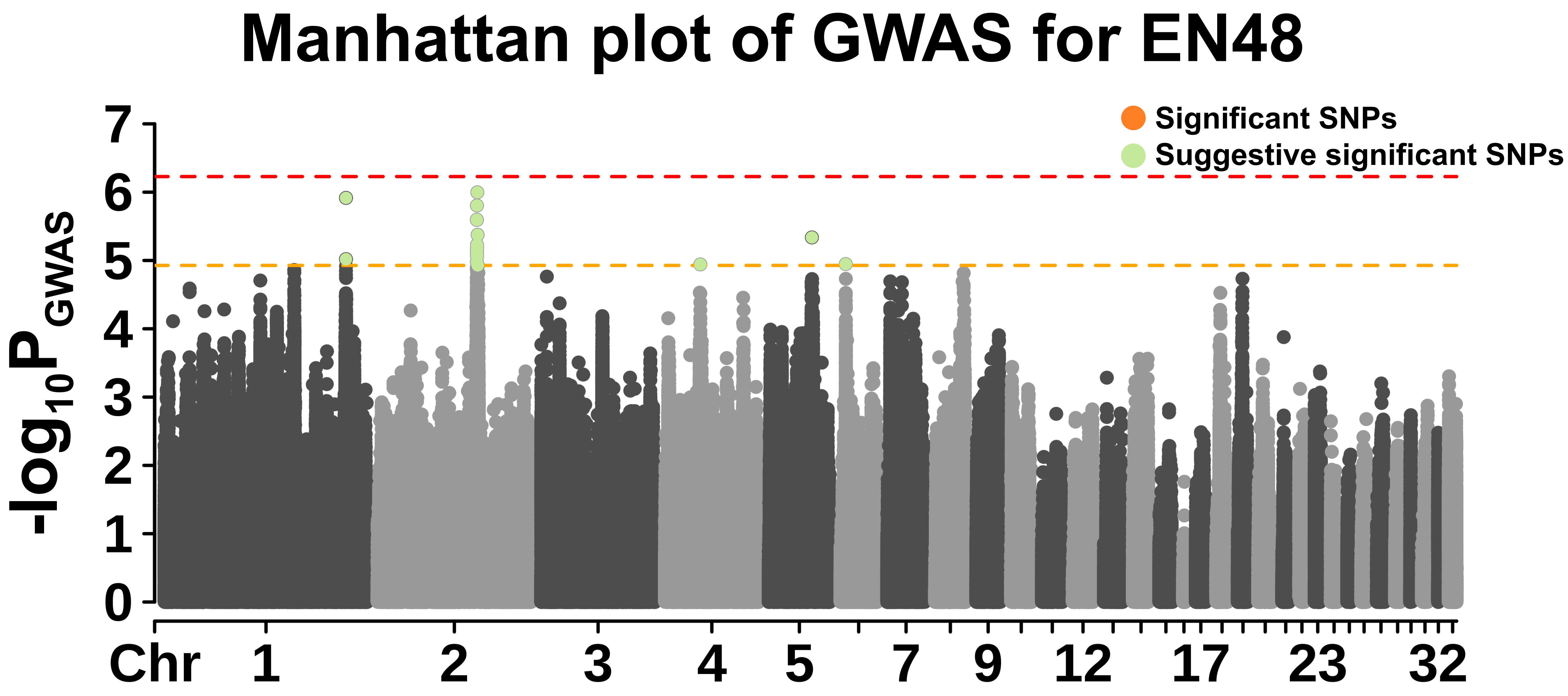

Manhattan plot of GWAS for EN48
Significant SNPs
Suggestive significant SNPs
-log10PGWAS
0
1
2
3
4
5
6
7
Chr
1
2
3
4
5
7
9
12
17
23
32

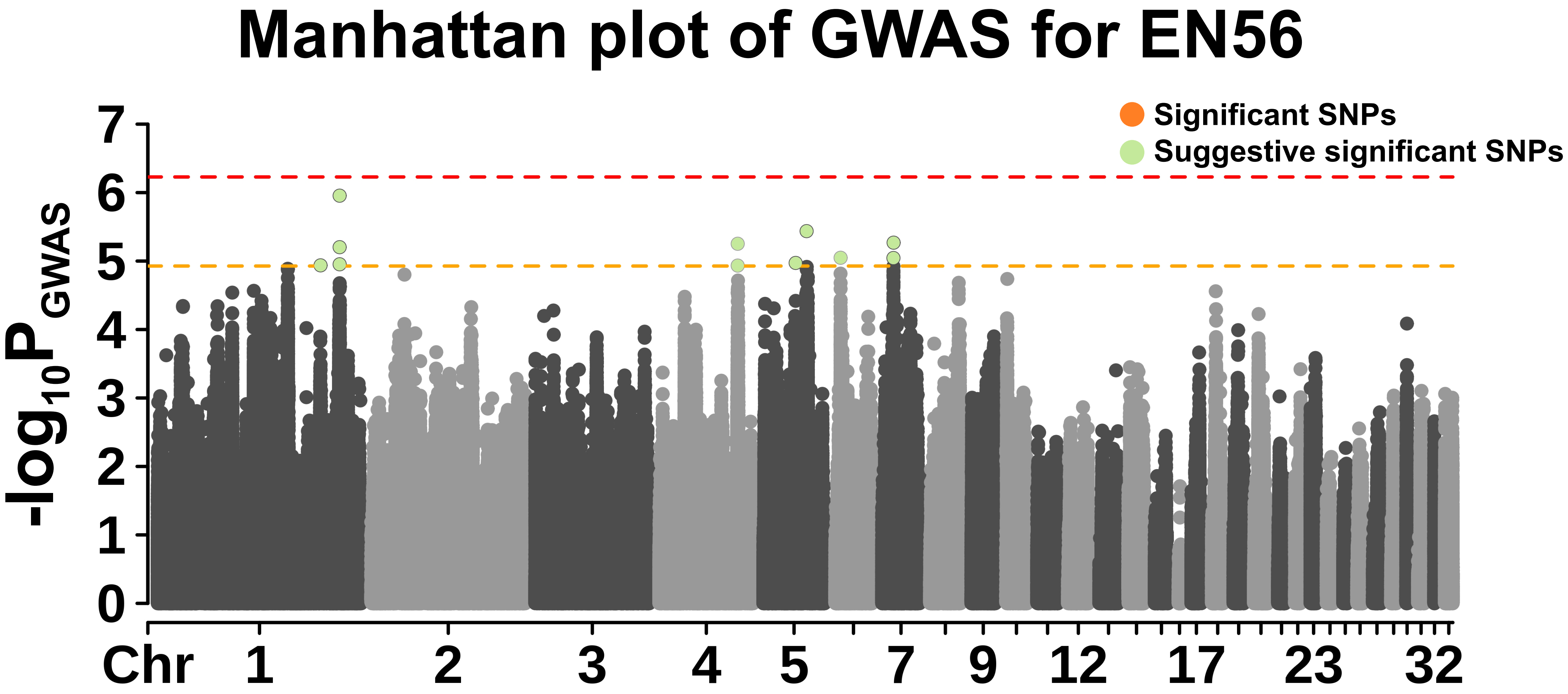
Manhattan plot of GWAS for EN56
Significant SNPs
Suggestive significant SNPs
-log10PGWAS
7
6
5
4
3
2
1
0
Chr
1
2
3
4
5
7
9
12
17
23
32

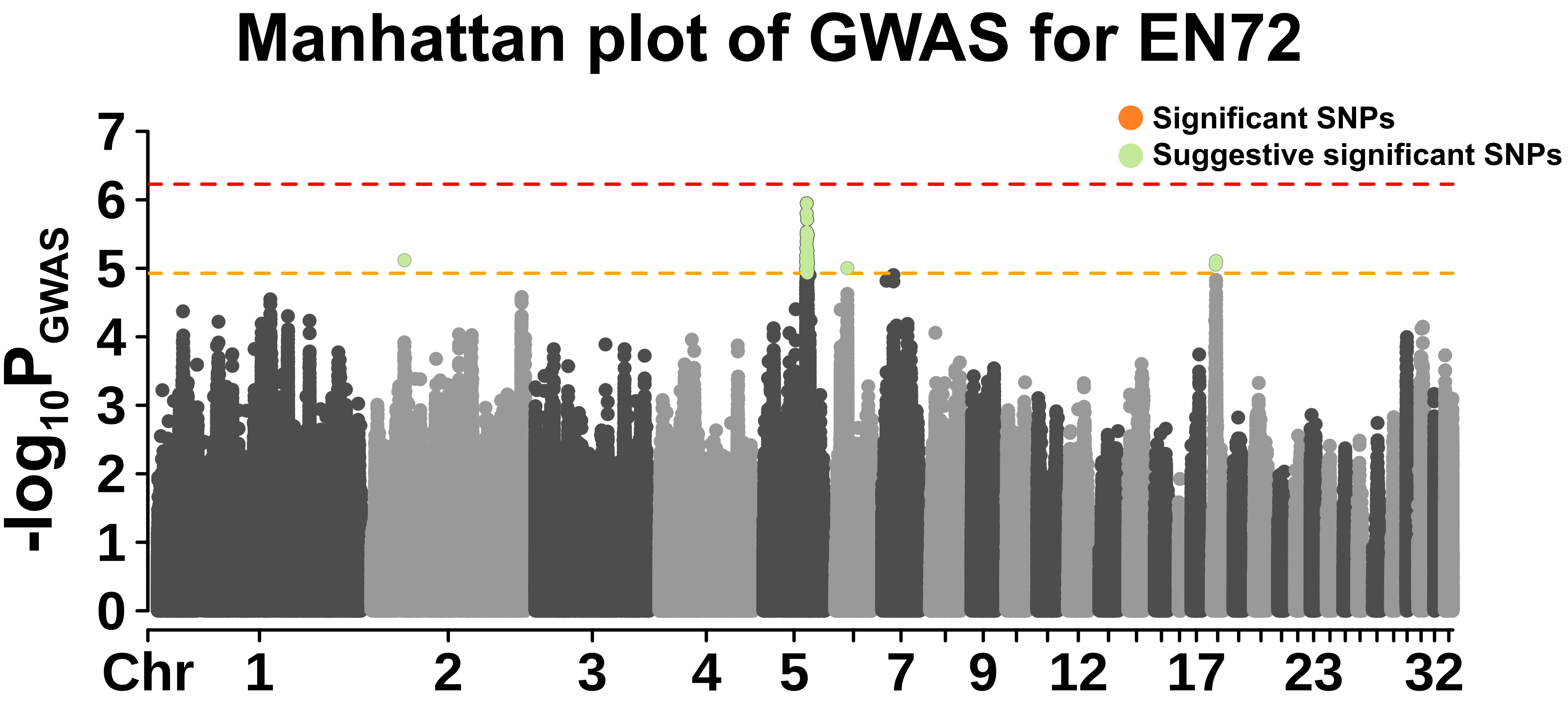

Manhattan plot of GWAS for EN72
Significant SNPs
Suggestive significant SNPs
$-\log_{10}P_{GWAS}$
0
1
2
3
4
5
6
7
Chr
1
2
3
4
5
7
9
12
17
23
32

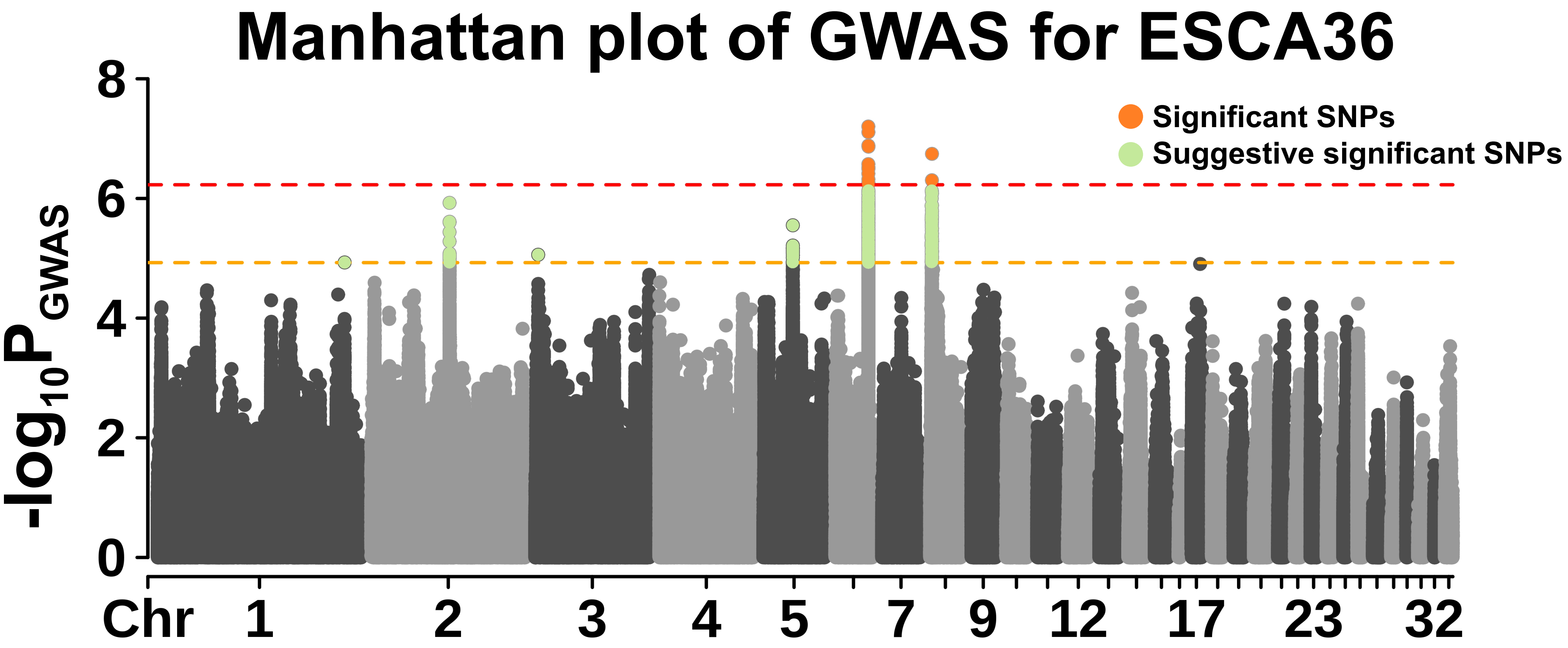
Manhattan plot of GWAS for ESCA36
Significant SNPs
Suggestive significant SNPs
-log10PGWAS
8
6
4
2
0
Chr
1
2
3
4
5
7
9
12
17
23
32

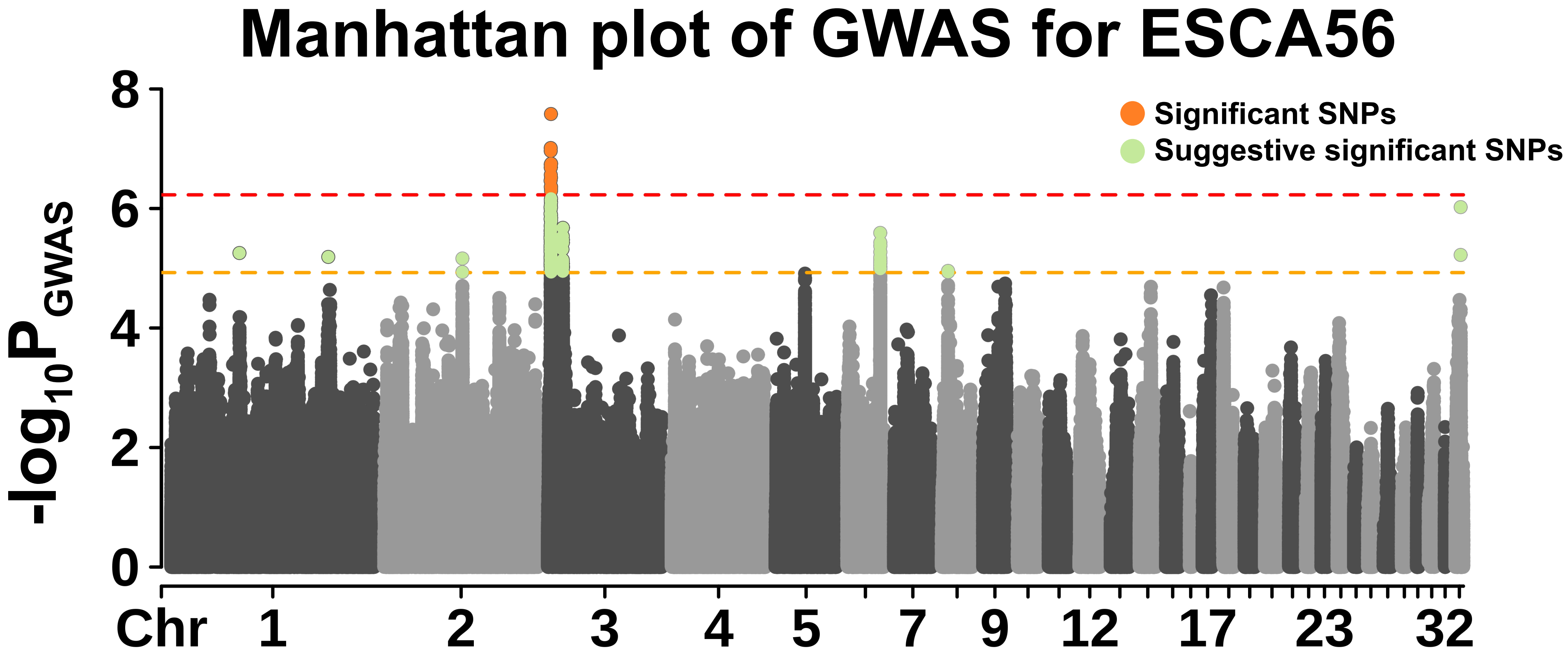

Manhattan plot of GWAS for ESCA56
Significant SNPs
Suggestive significant SNPs
-log10PGWAS
0
2
4
6
8
Chr
1
2
3
4
5
7
9
12
17
23
32

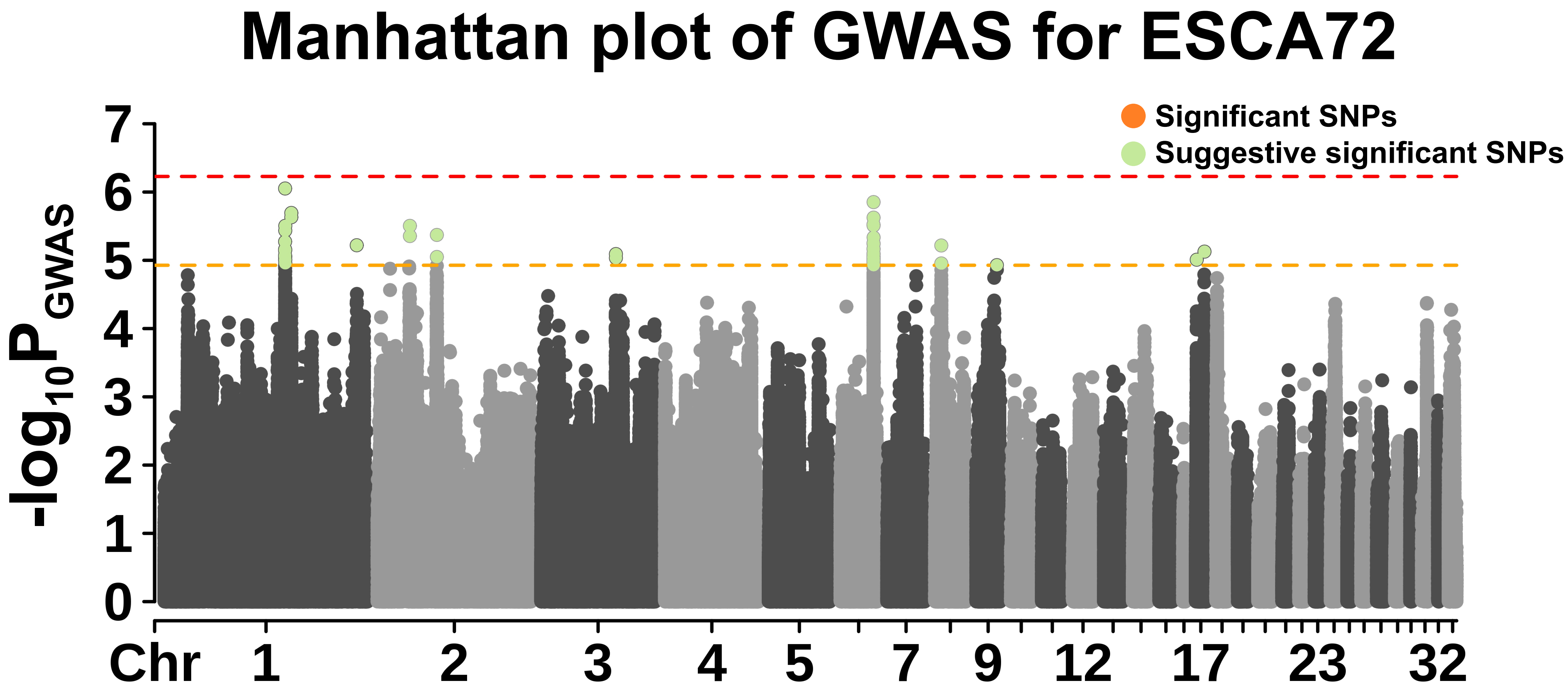
Manhattan plot of GWAS for ESCA72
Significant SNPs
Suggestive significant SNPs
-log10PGWAS
0
1
2
3
4
5
6
7
Chr
1
2
3
4
5
7
9
12
17
23
32

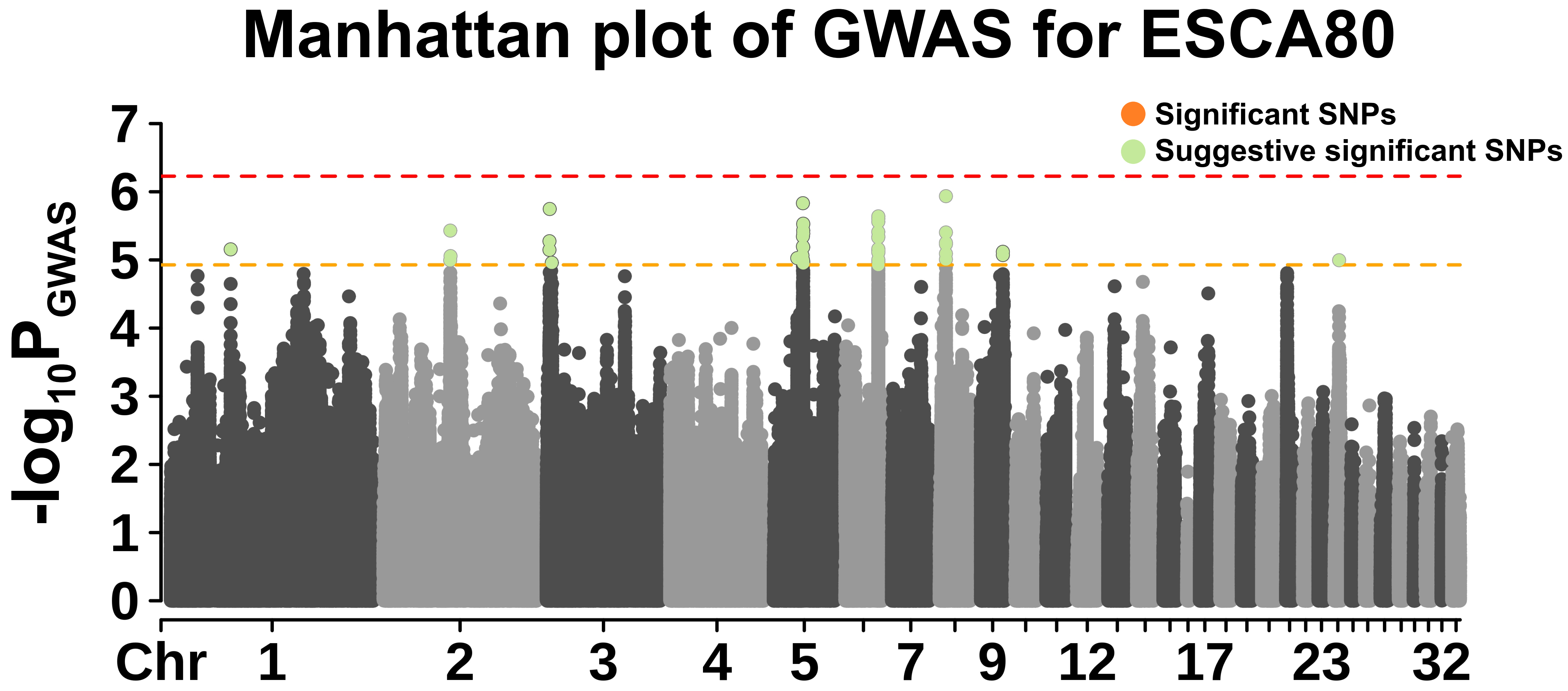

Manhattan plot of GWAS for ESCA80
Significant SNPs
Suggestive significant SNPs
$-log_{10}P_{GWAS}$
0
1
2
3
4
5
6
7
Chr
1
2
3
4
5
7
9
12
17
23
32

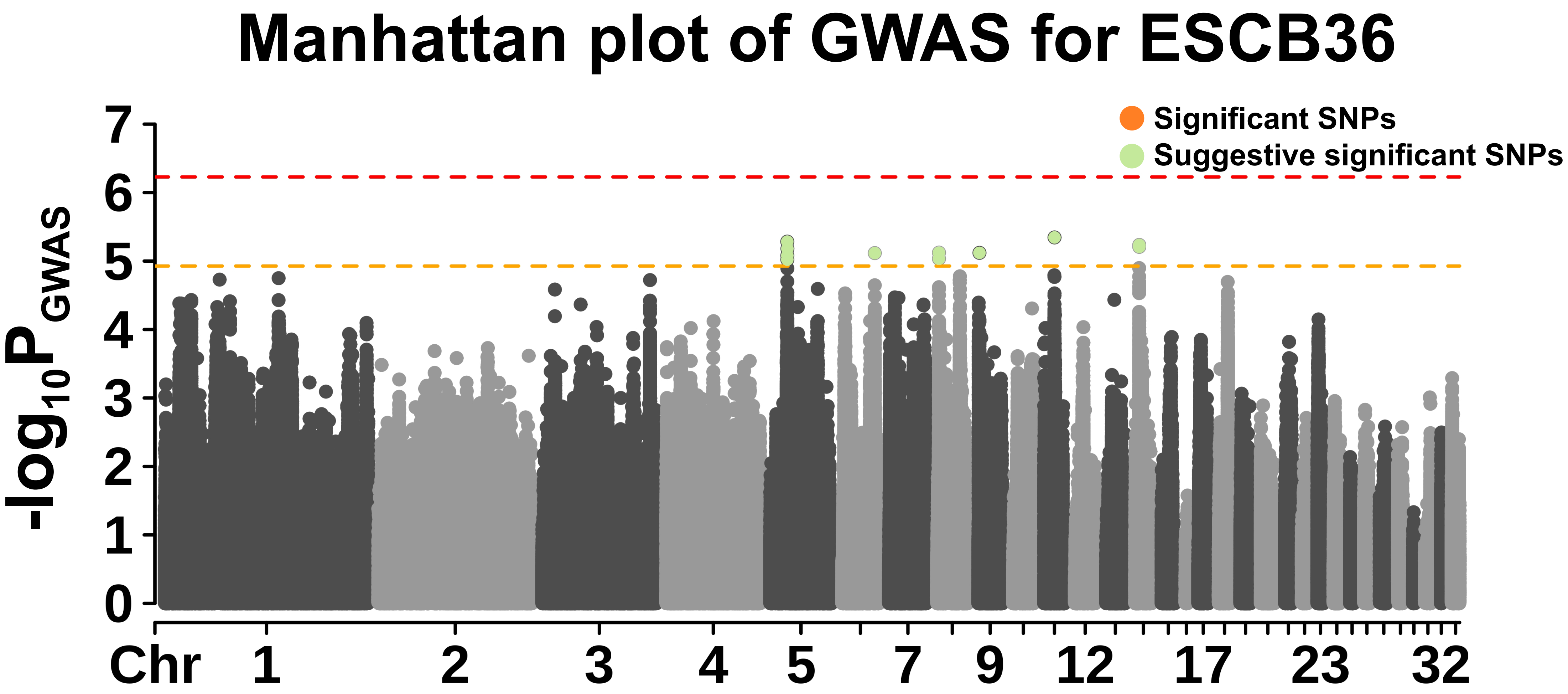

Manhattan plot of GWAS for ESCB36
Significant SNPs
Suggestive significant SNPs
$-\log_{10}P_{GWAS}$
0
1
2
3
4
5
6
7
Chr
1
2
3
4
5
7
9
12
17
23
32

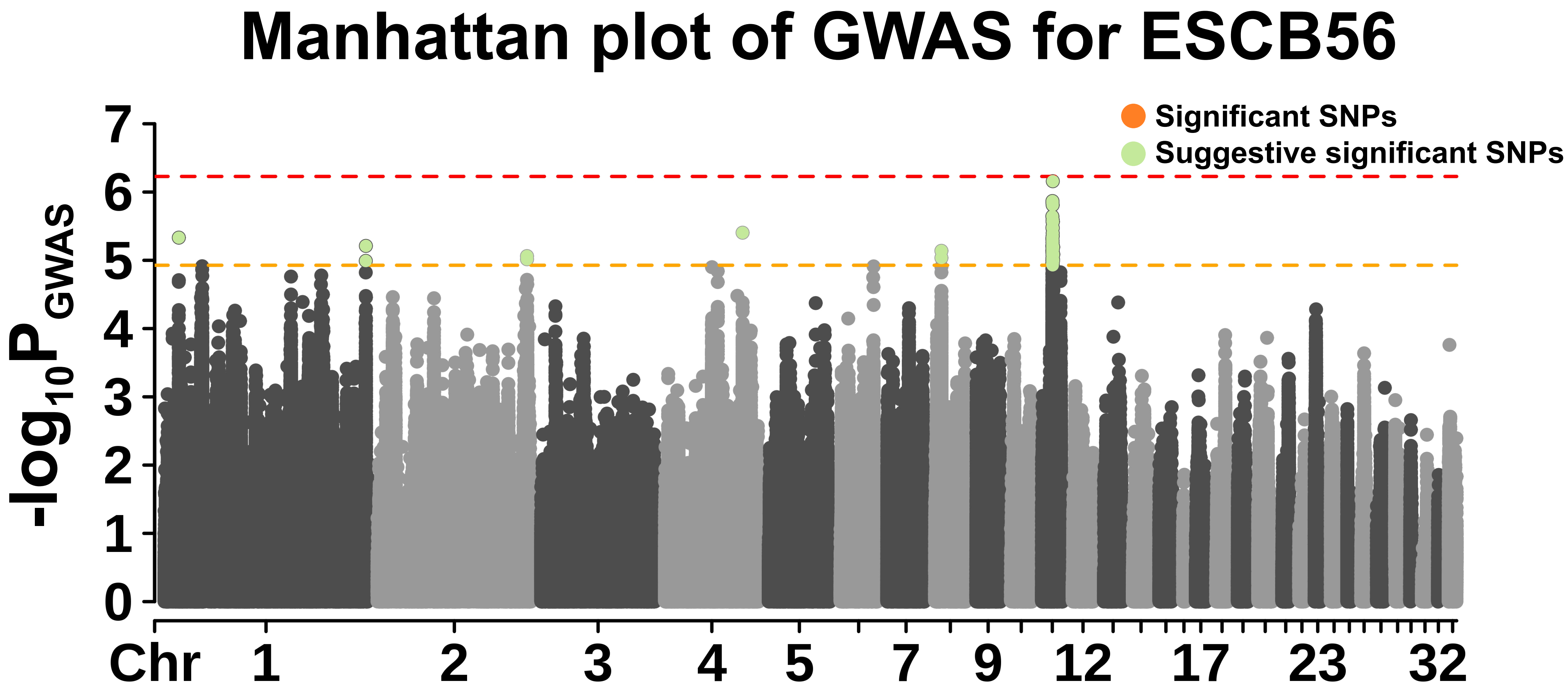

Manhattan plot of GWAS for ESCB56
Significant SNPs
Suggestive significant SNPs
-log10PGWAS
0
1
2
3
4
5
6
7
Chr
1
2
3
4
5
7
9
12
17
23
32

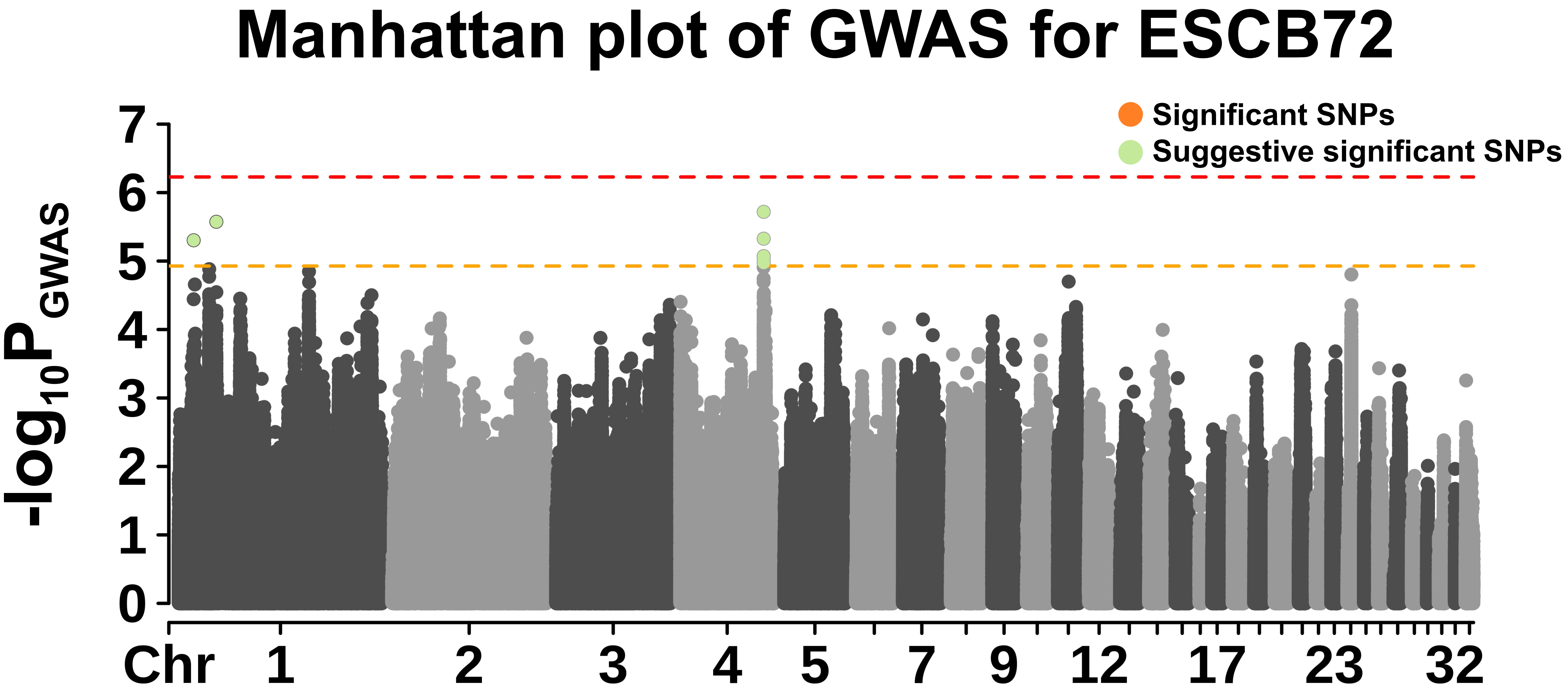
Manhattan plot of GWAS for ESCB72
Significant SNPs
Suggestive significant SNPs
-log10PGWAS
0
1
2
3
4
5
6
7
Chr
1
2
3
4
5
7
9
12
17
23
32

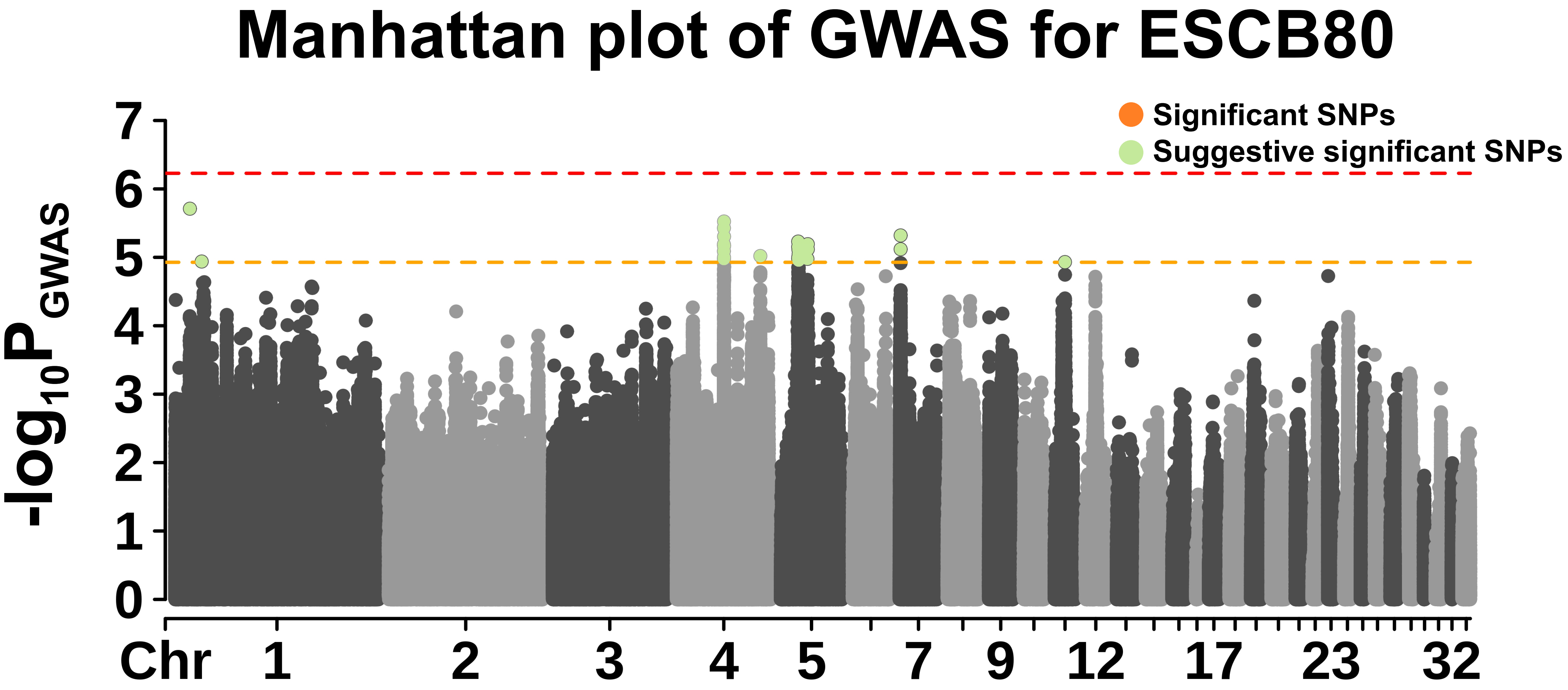

Manhattan plot of GWAS for ESCB80
Significant SNPs
Suggestive significant SNPs
-log10PGWAS
0
1
2
3
4
5
6
7
Chr
1
2
3
4
5
7
9
12
17
23
32

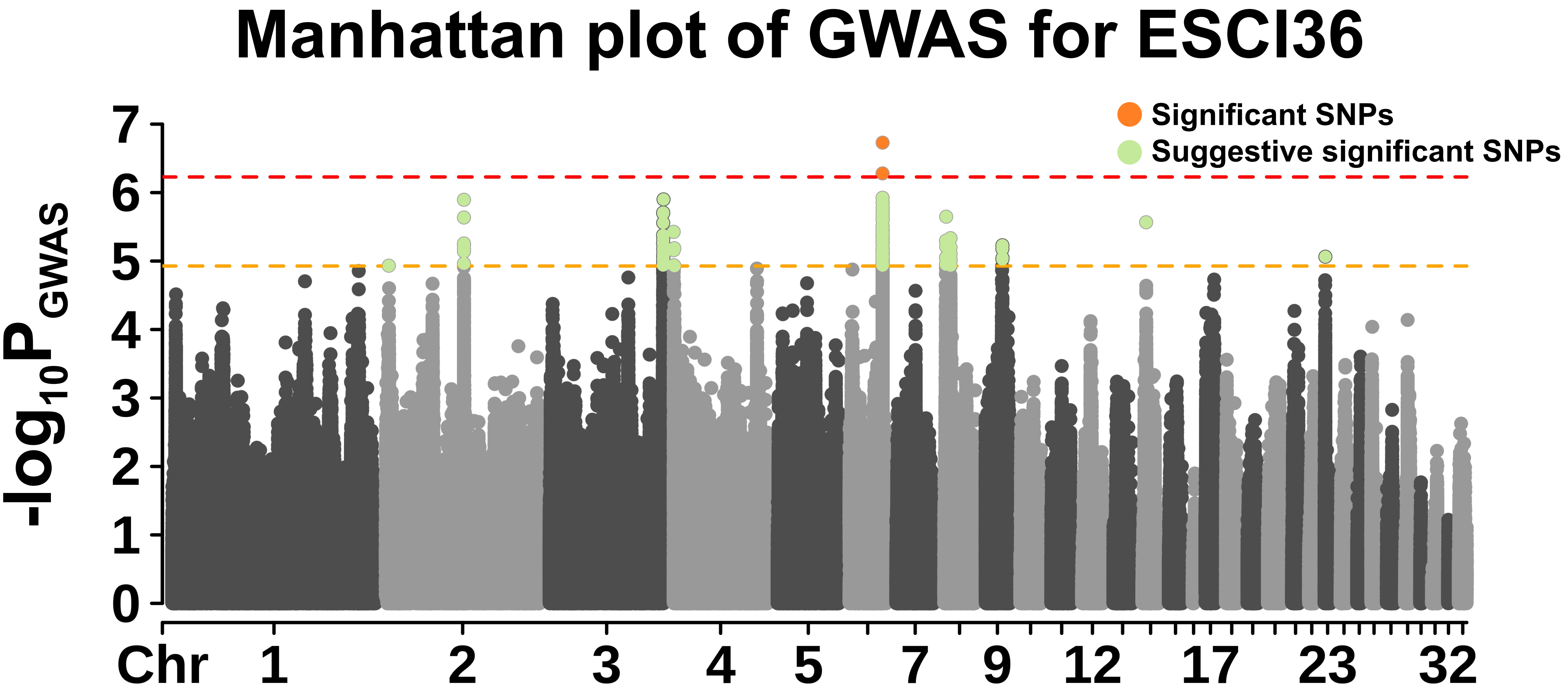

Manhattan plot of GWAS for ESCI36
Significant SNPs
Suggestive significant SNPs
-log10PGWAS
0
1
2
3
4
5
6
7
Chr
1
2
3
4
5
7
9
12
17
23
32

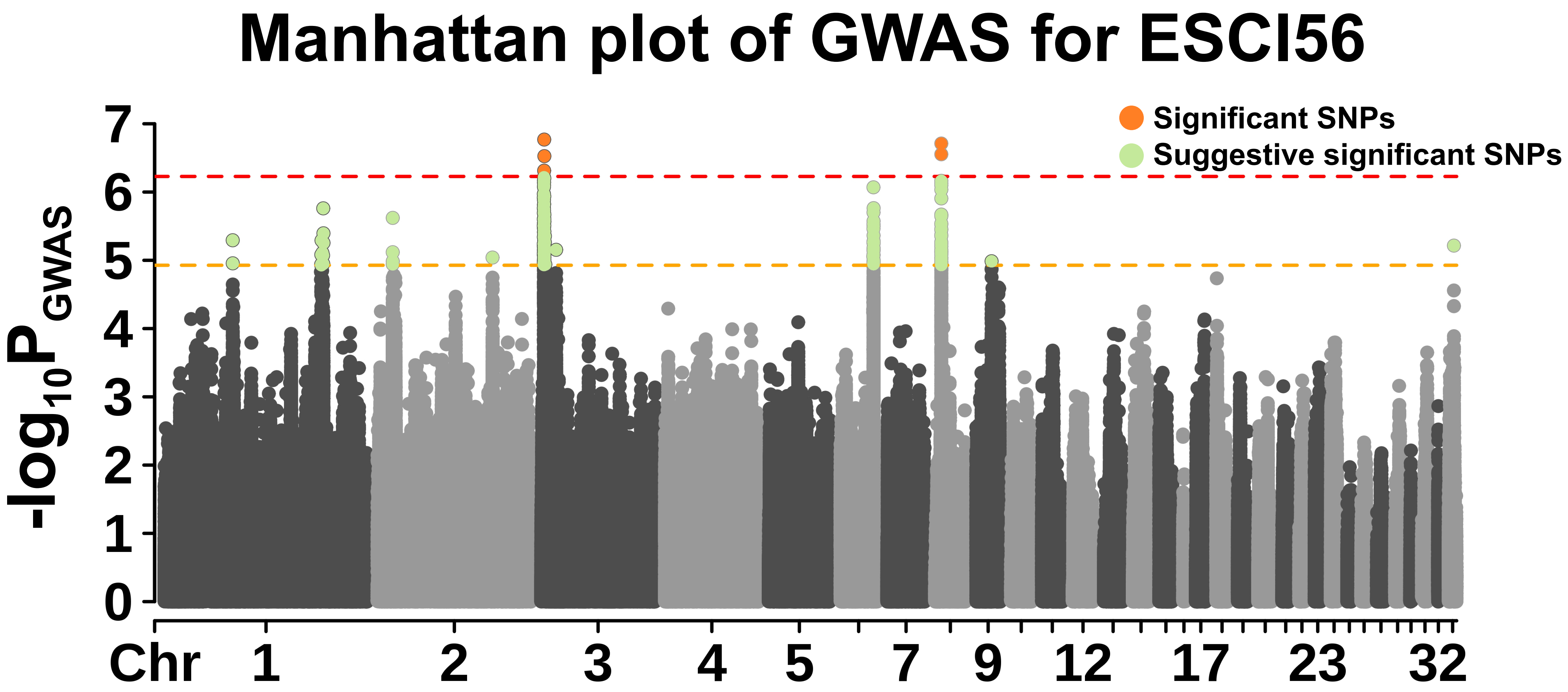
Manhattan plot of GWAS for ESCI56
Significant SNPs
Suggestive significant SNPs
-log10PGWAS
0
1
2
3
4
5
6
7
Chr
1
2
3
4
5
7
9
12
17
23
32

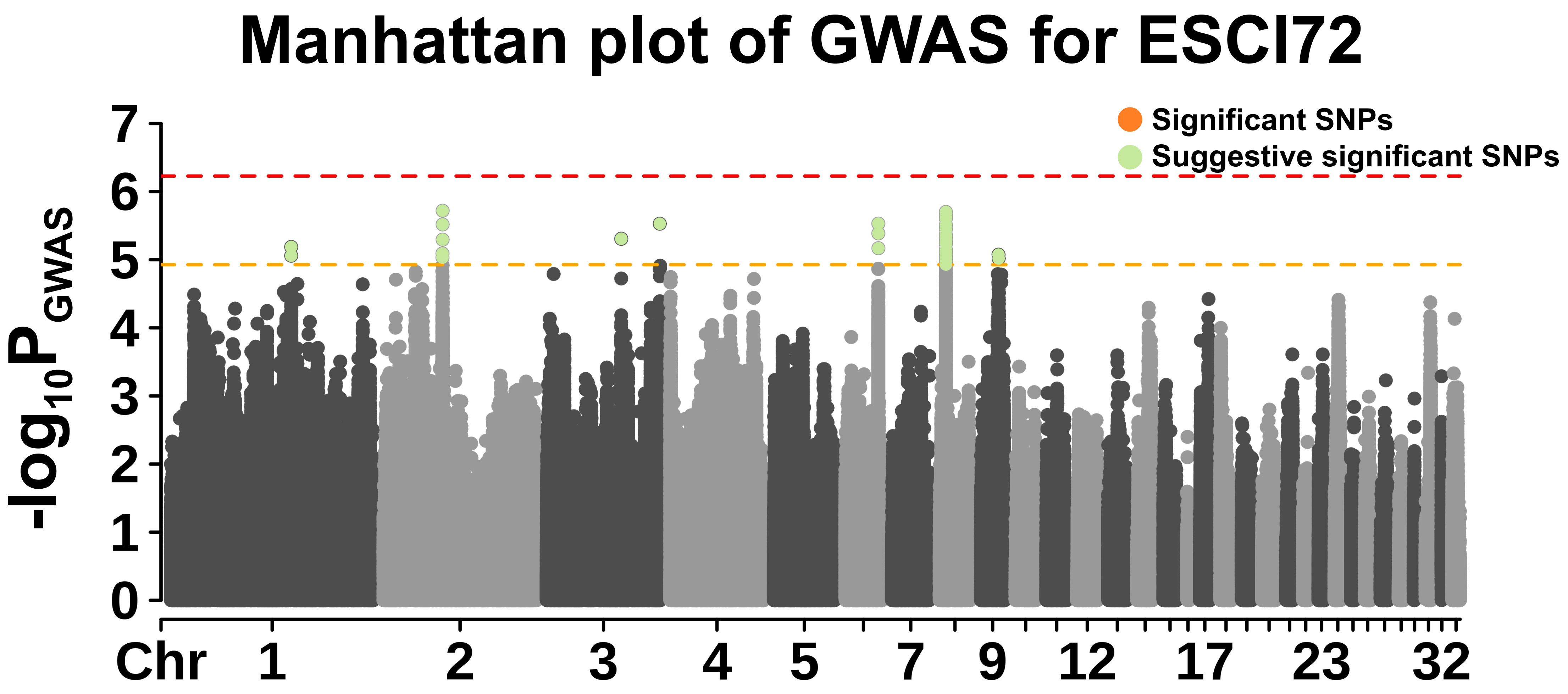
Manhattan plot of GWAS for ESCI72
Significant SNPs
Suggestive significant SNPs
$-\log_{10}P_{GWAS}$
0
1
2
3
4
5
6
7
Chr
1
2
3
4
5
7
9
12
17
23
32

Manhattan plot of GWAS for ESCI80

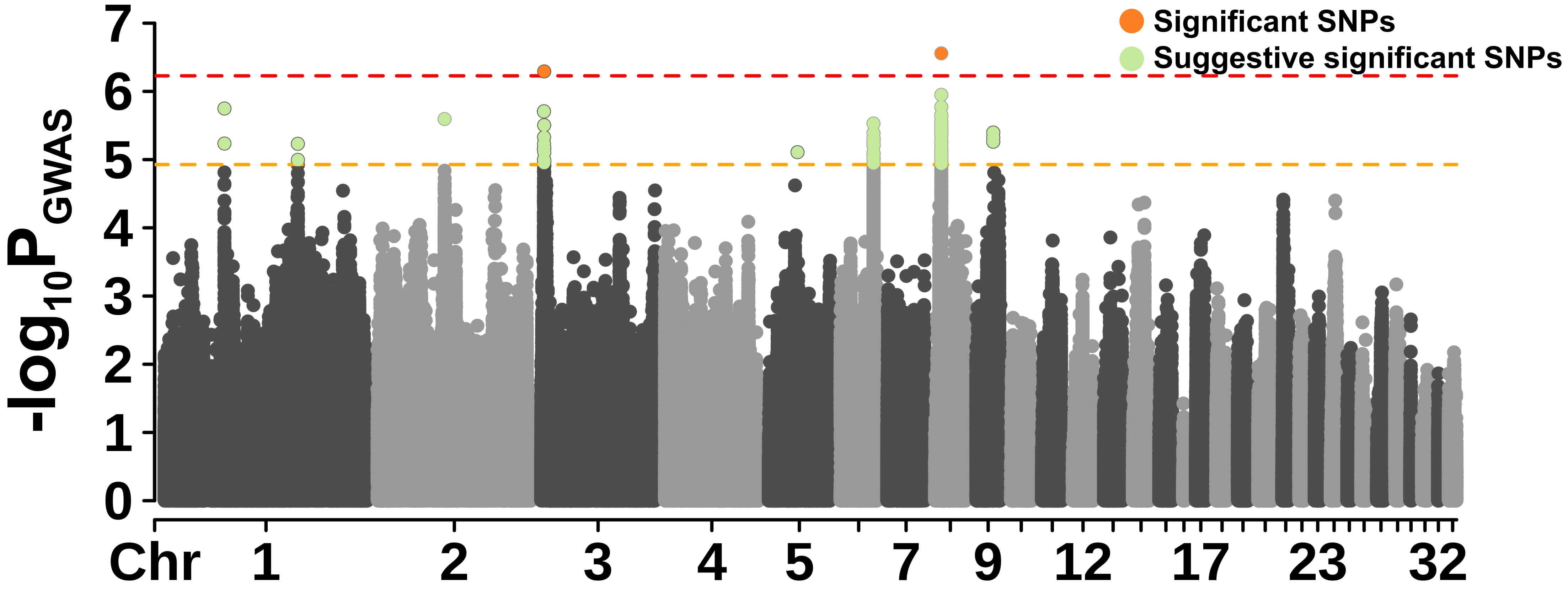

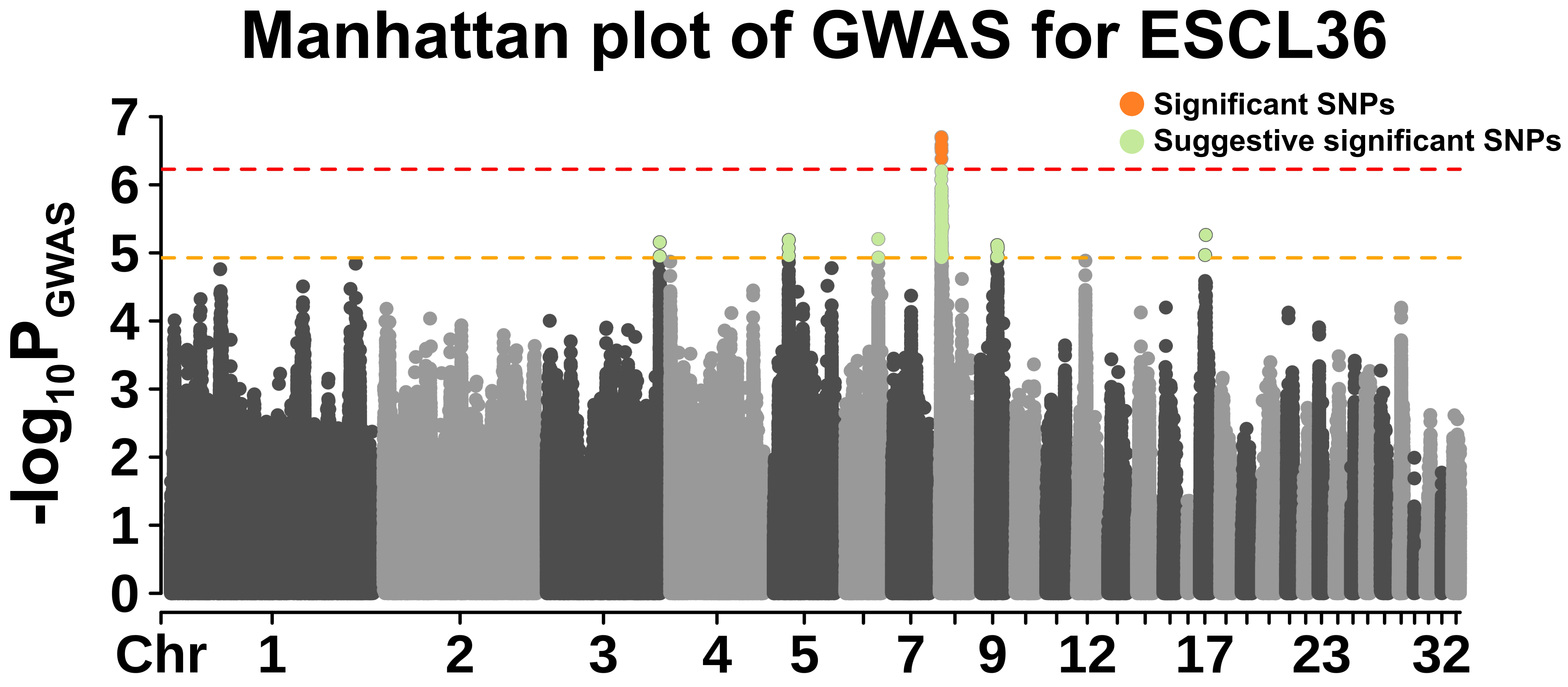

Manhattan plot of GWAS for ESCL36
Significant SNPs
Suggestive significant SNPs
$-\log_{10}P_{GWAS}$
0
1
2
3
4
5
6
7
Chr
1
2
3
4
5
7
9
12
17
23
32

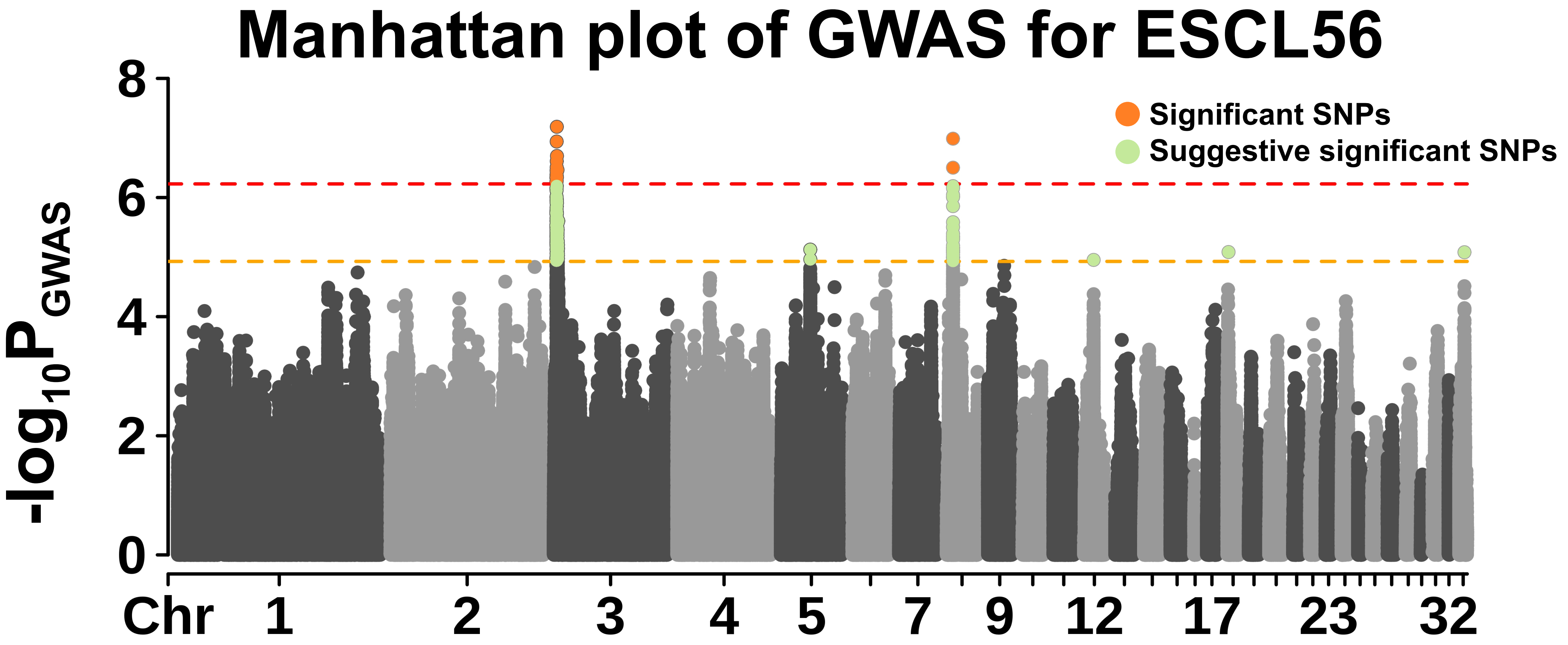

Manhattan plot of GWAS for ESCL56
Significant SNPs
Suggestive significant SNPs
$-log_{10}P_{GWAS}$
8
6
4
2
0
Chr
1
2
3
4
5
7
9
12
17
23
32

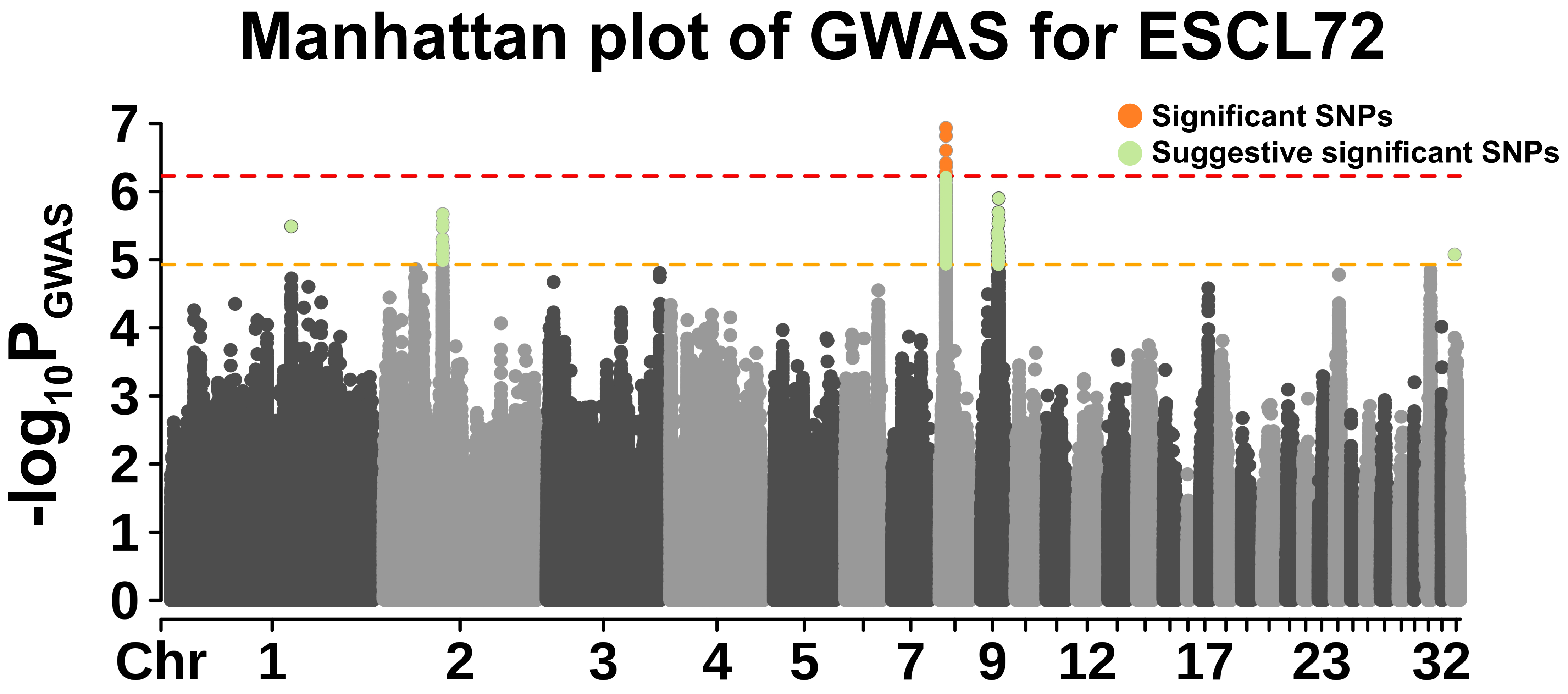
Manhattan plot of GWAS for ESCL72
Significant SNPs
Suggestive significant SNPs
$-\log_{10}P_{GWAS}$
0
1
2
3
4
5
6
7
Chr
1
2
3
4
5
7
9
12
17
23
32

Manhattan plot of GWAS for ESCL80

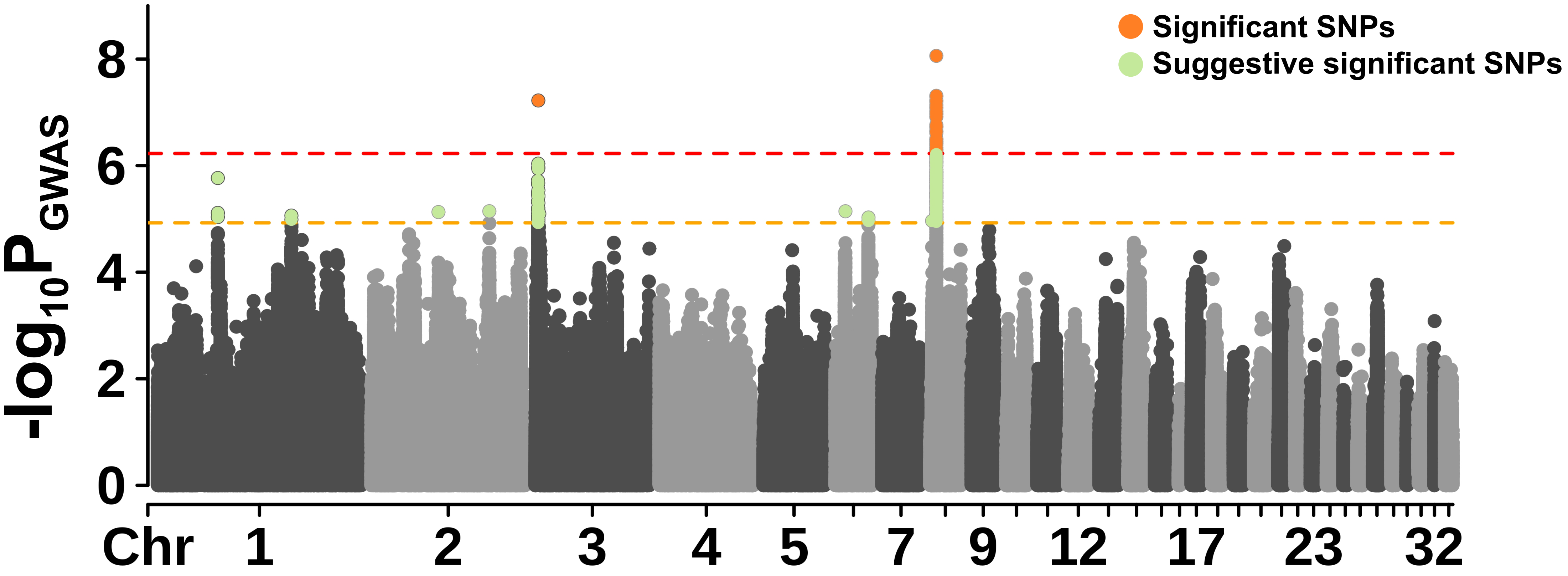

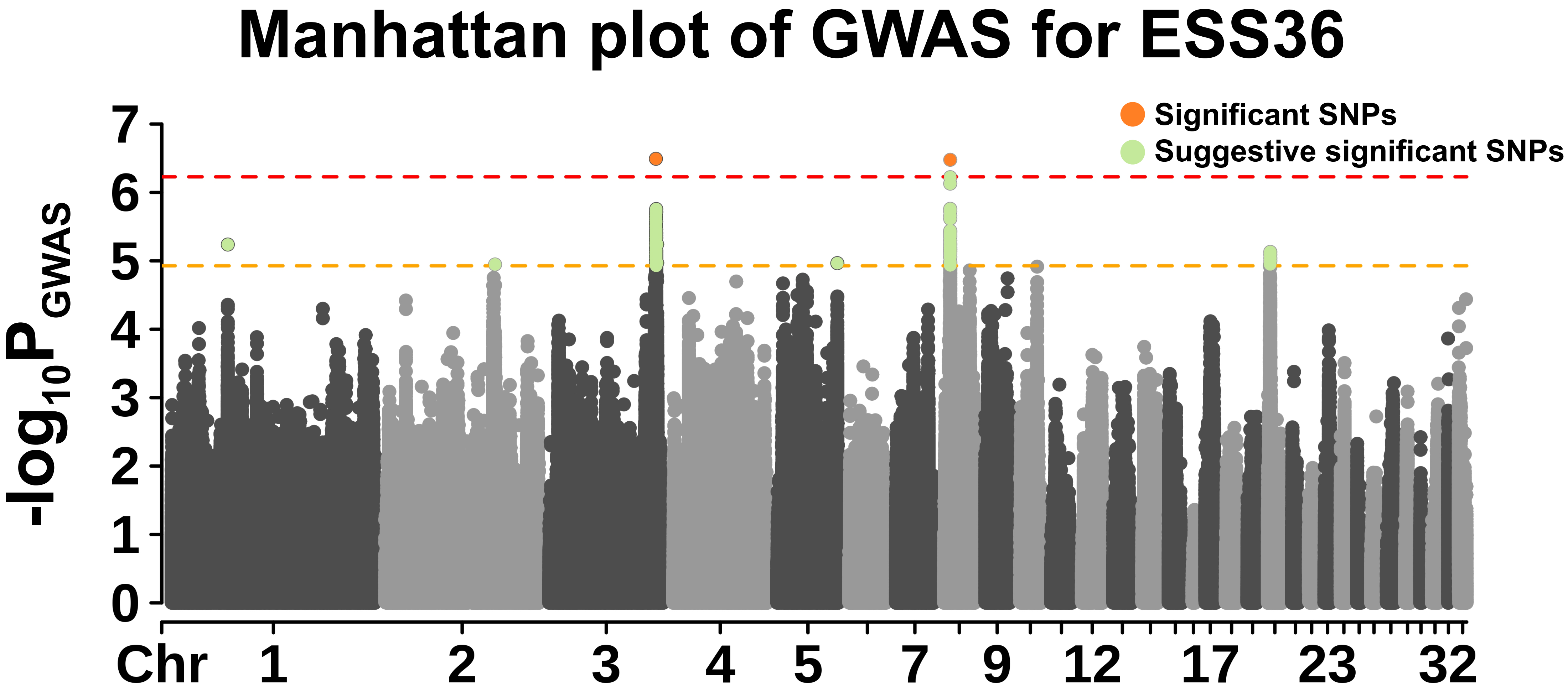
Manhattan plot of GWAS for ESS36
Significant SNPs
Suggestive significant SNPs
$-\log_{10}P_{GWAS}$
0
1
2
3
4
5
6
7
Chr
1
2
3
4
5
7
9
12
17
23
32

Manhattan plot of GWAS for ESS56

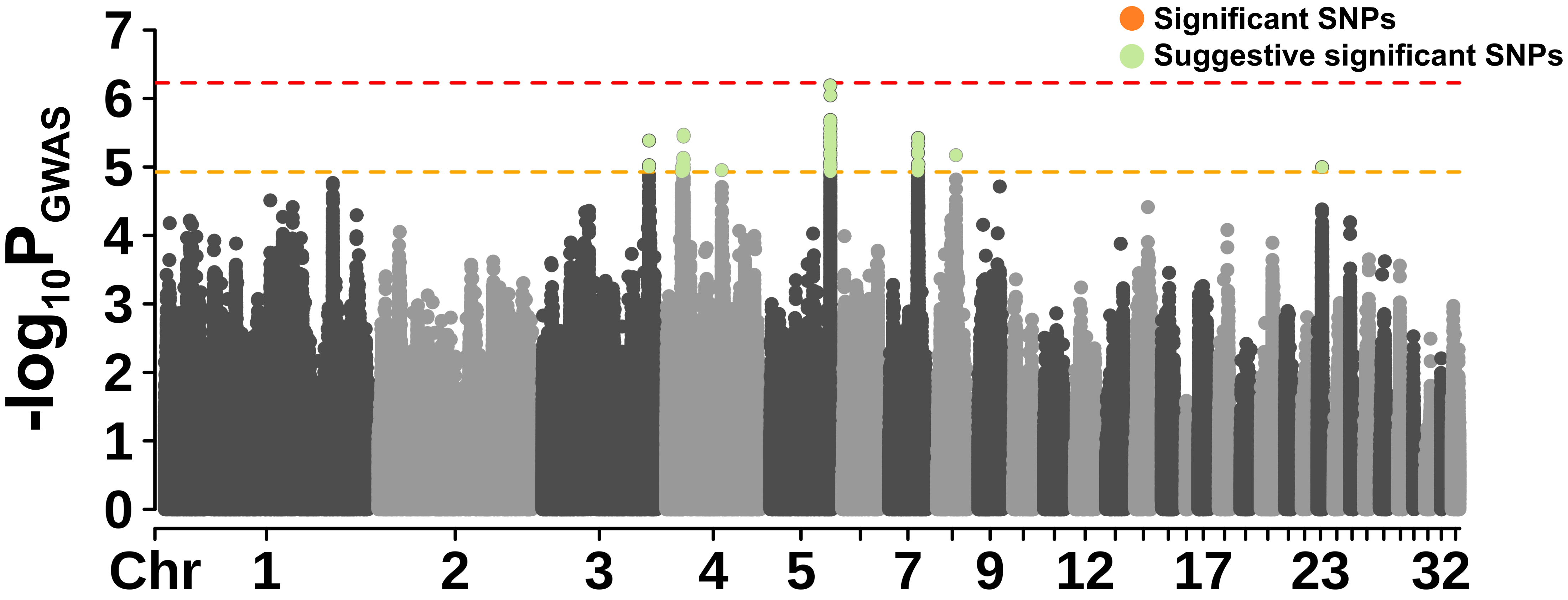

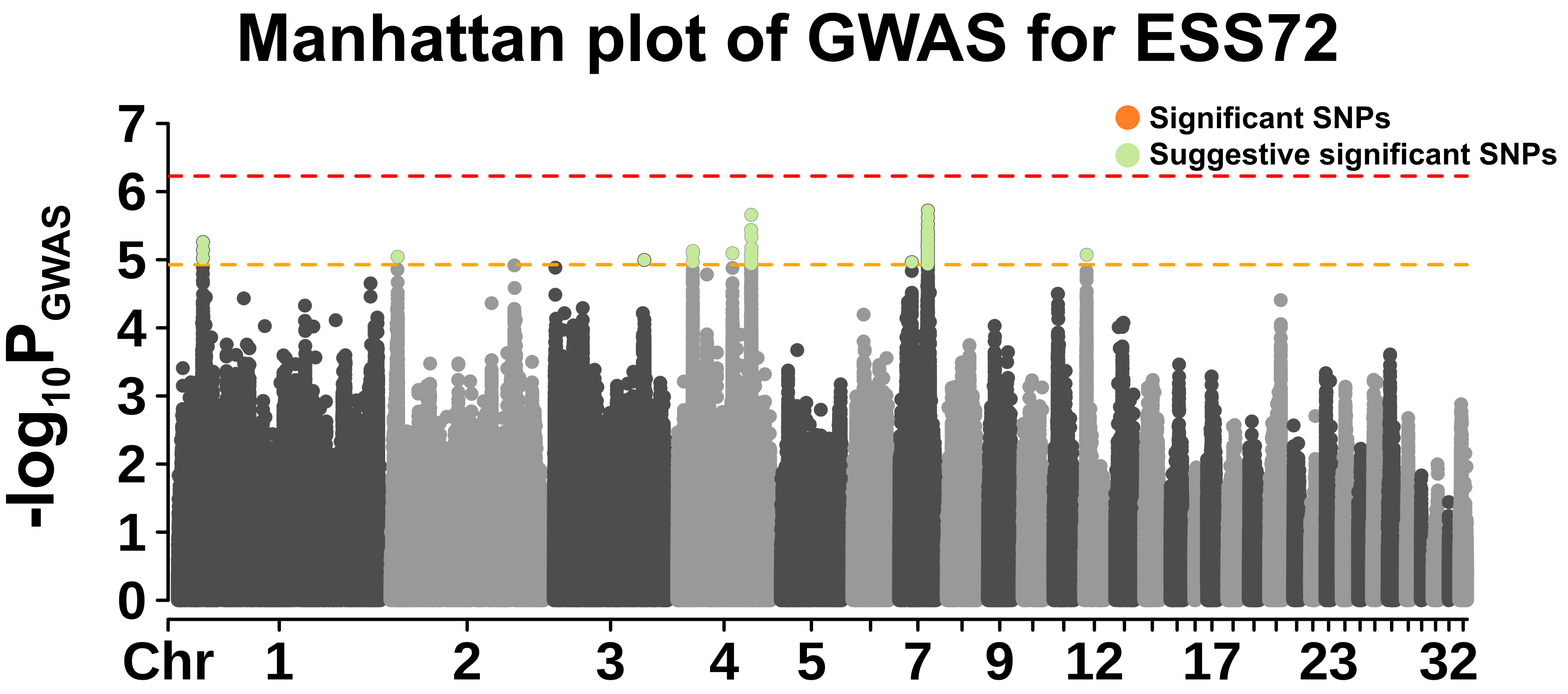
Manhattan plot of GWAS for ESS72
Significant SNPs
Suggestive significant SNPs
-log10PGWAS
7
6
5
4
3
2
1
0
Chr
1
2
3
4
5
7
9
12
17
23
32

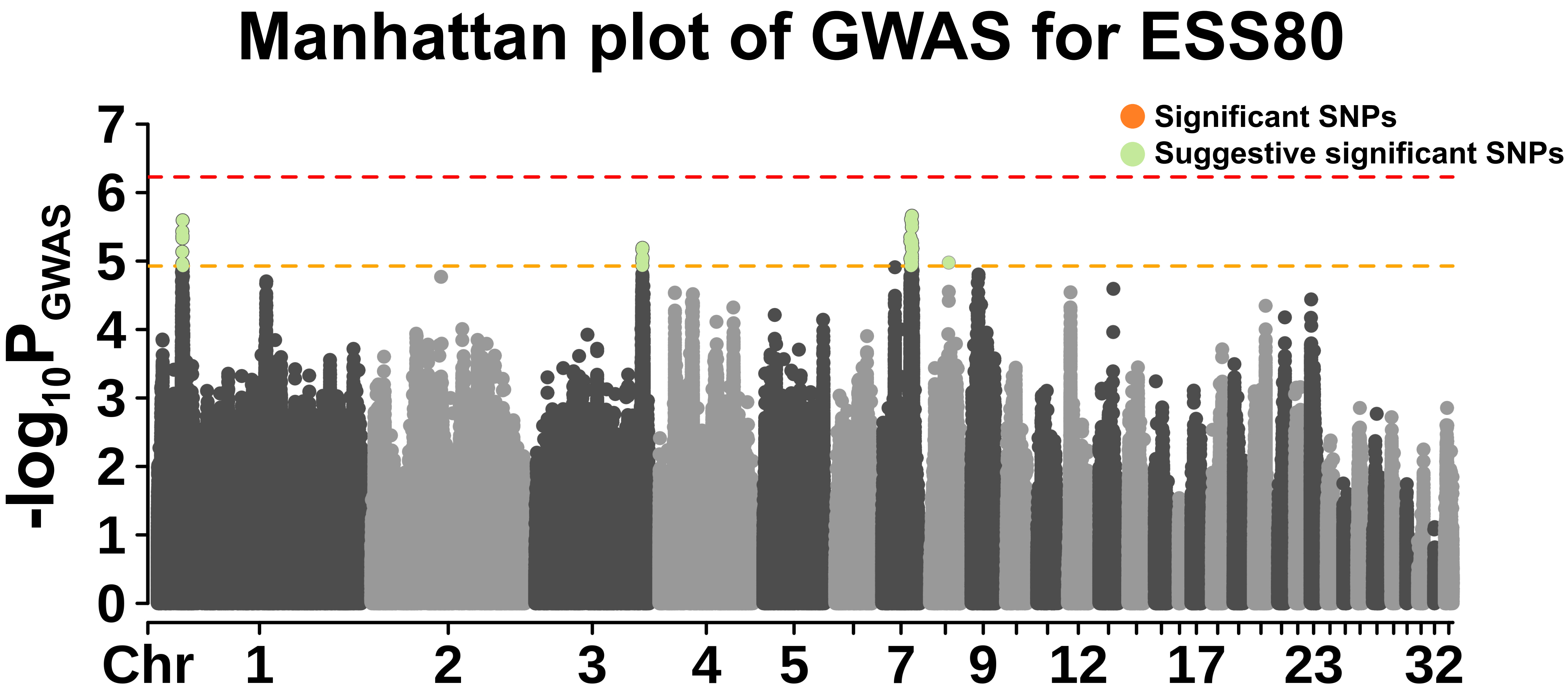

Manhattan plot of GWAS for ESS80
Significant SNPs
Suggestive significant SNPs
$-\log_{10}P_{GWAS}$
0
1
2
3
4
5
6
7
Chr
1
2
3
4
5
7
9
12
17
23
32

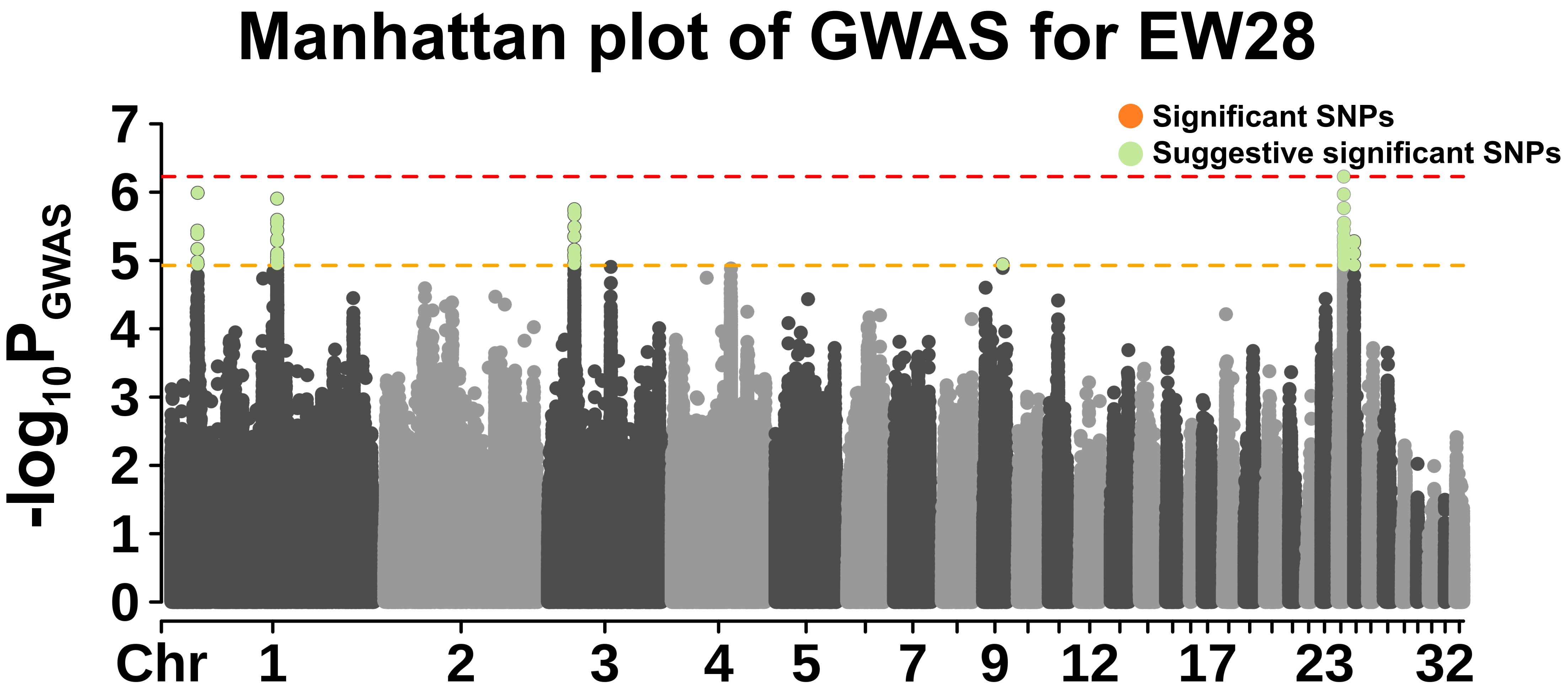

Manhattan plot of GWAS for EW28
Significant SNPs
Suggestive significant SNPs
$-\log_{10}P_{GWAS}$
0
1
2
3
4
5
6
7
Chr
1
2
3
4
5
7
9
12
17
23
32

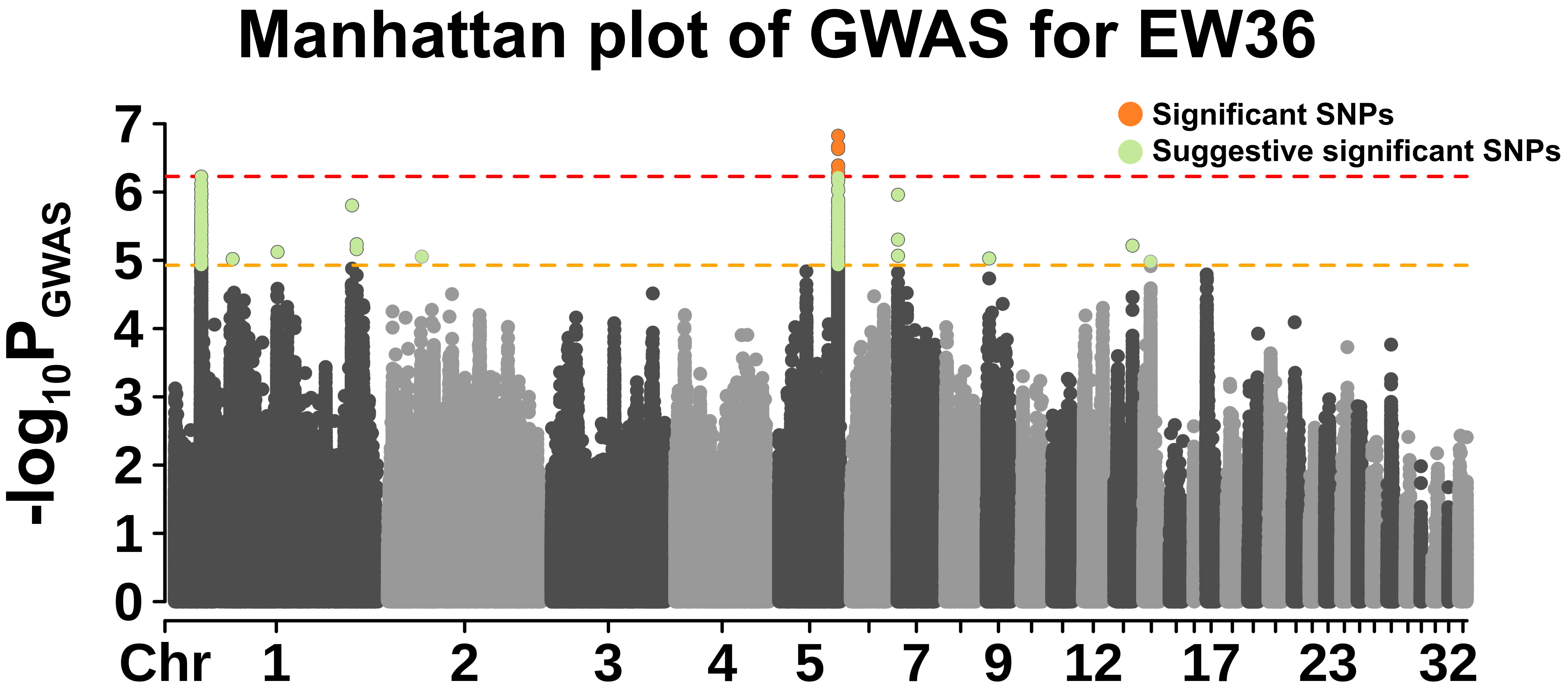

Manhattan plot of GWAS for EW36
Significant SNPs
Suggestive significant SNPs
-log10PGWAS
0
1
2
3
4
5
6
7
Chr
1
2
3
4
5
7
9
12
17
23
32

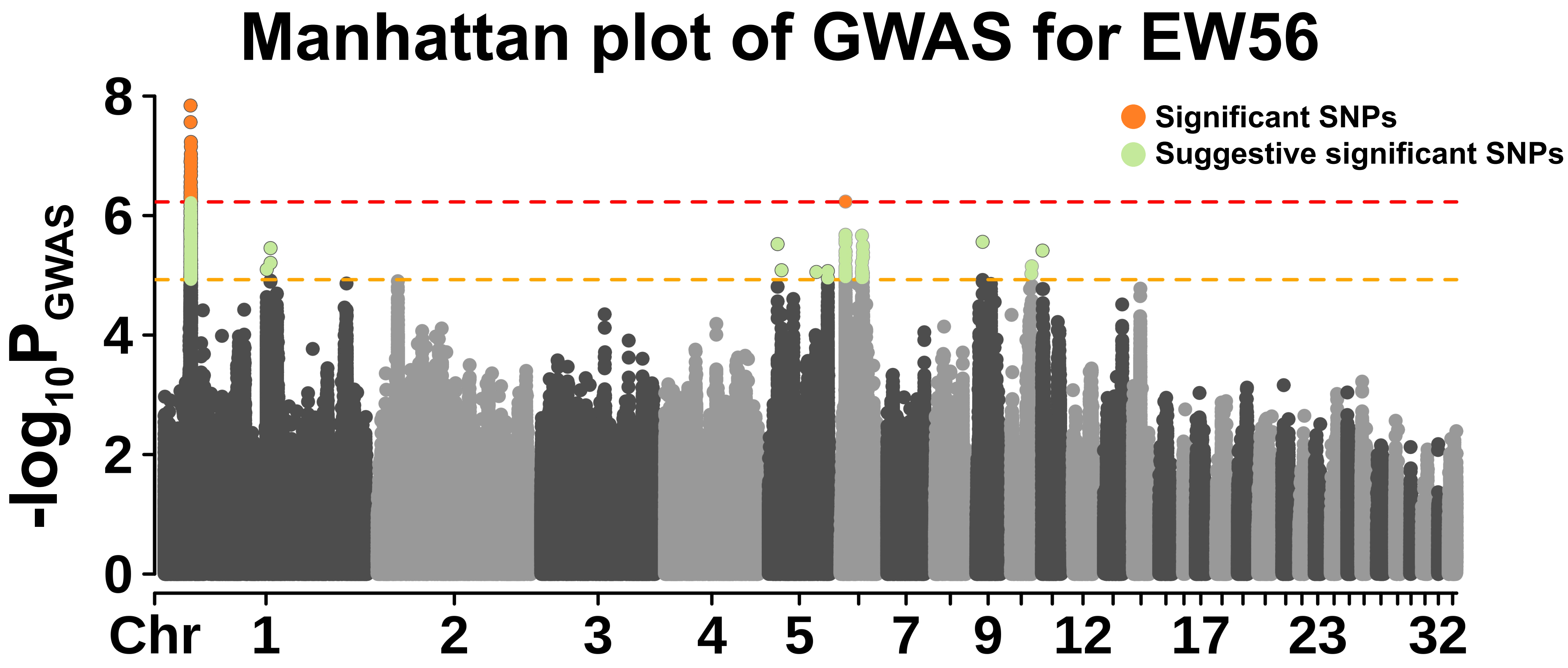
Manhattan plot of GWAS for EW56
Significant SNPs
Suggestive significant SNPs
$-\log_{10}P_{GWAS}$
0
2
4
6
8
Chr
1
2
3
4
5
7
9
12
17
23
32

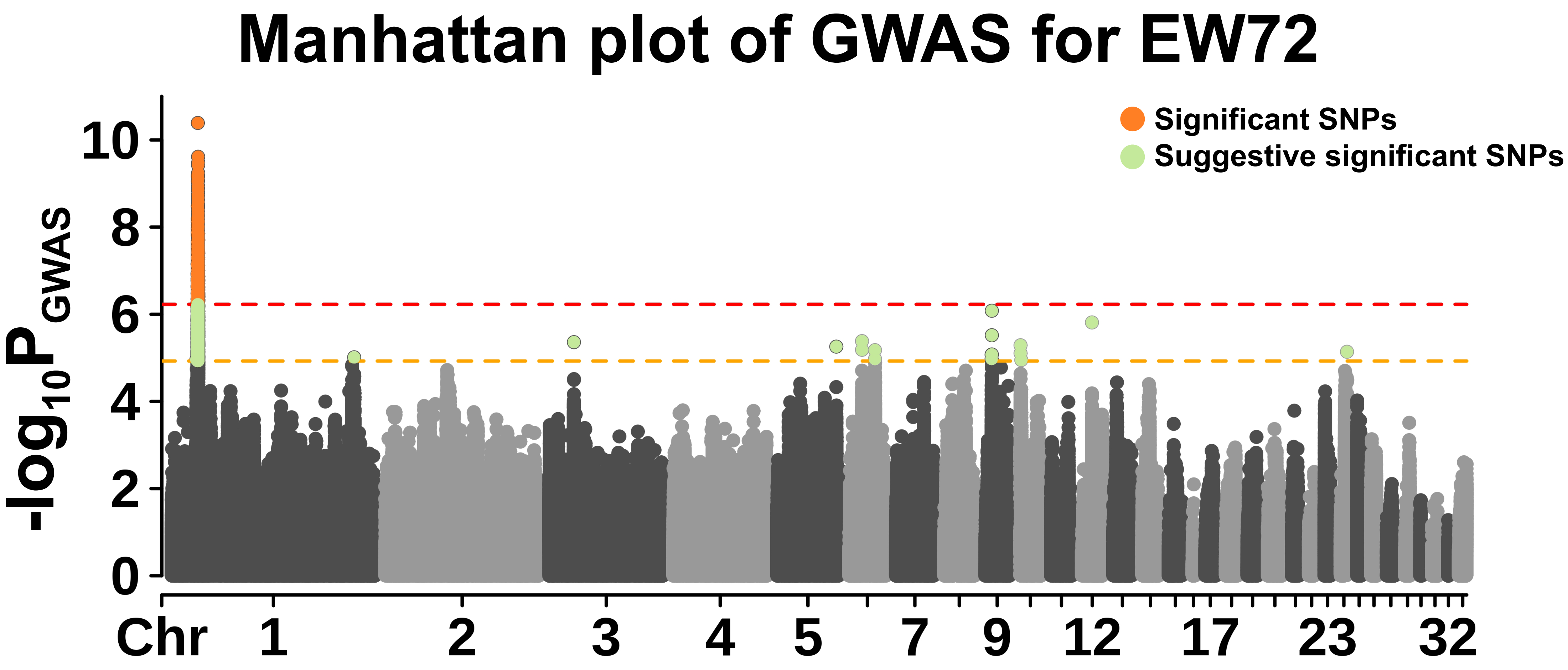

Manhattan plot of GWAS for EW72
Significant SNPs
Suggestive significant SNPs
-log10PGWAS
0
2
4
6
8
10
Chr
1
2
3
4
5
7
9
12
17
23
32

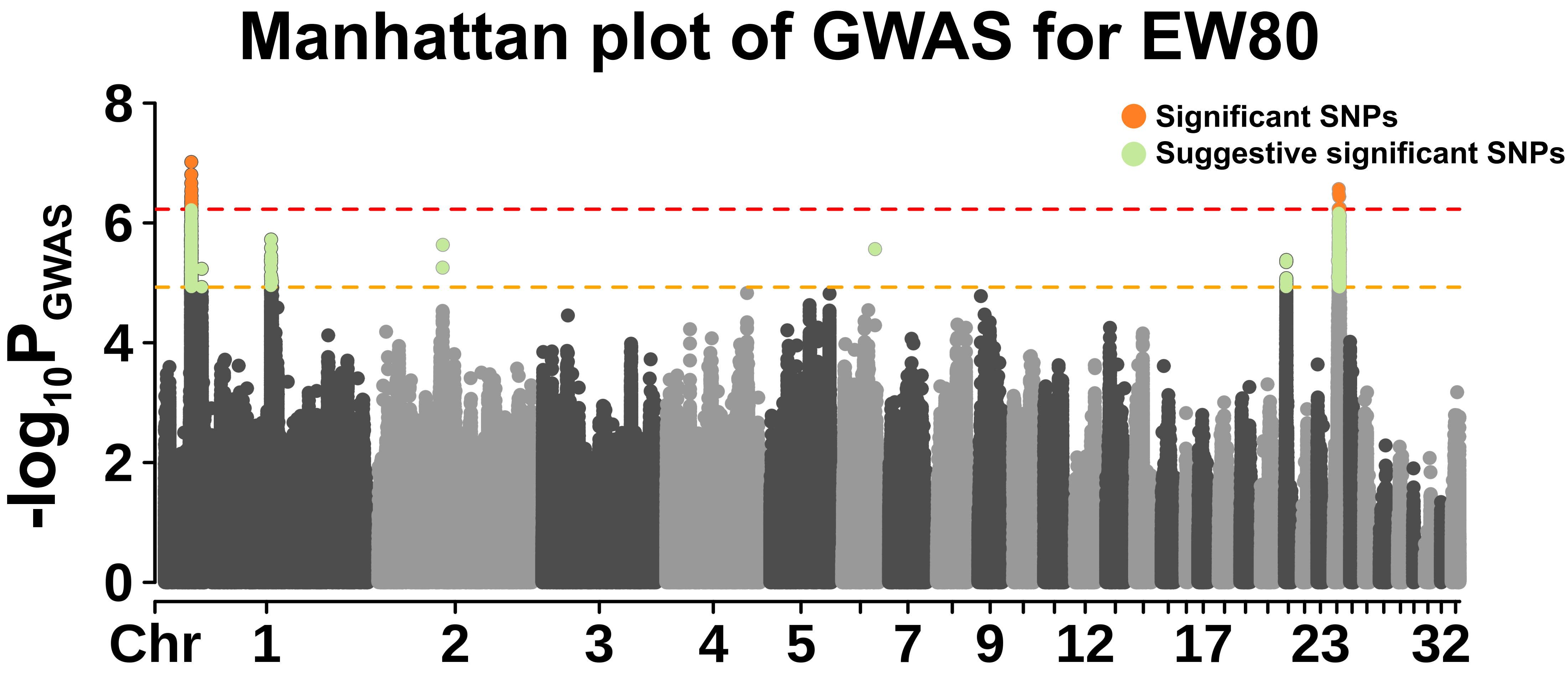

Manhattan plot of GWAS for EW80
Significant SNPs
Suggestive significant SNPs
-log10PGWAS
8
6
4
2
0
Chr
1
2
3
4
5
7
9
12
17
23
32

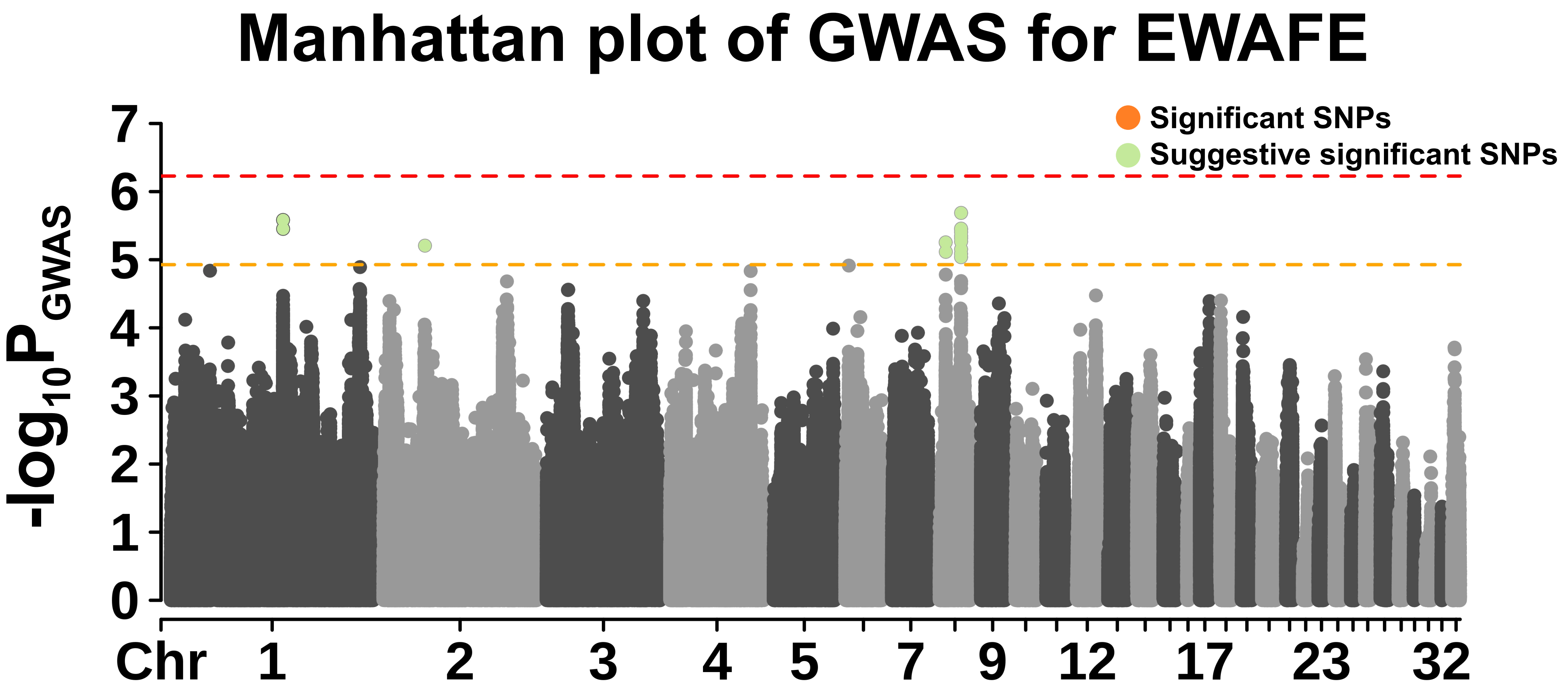

Manhattan plot of GWAS for EWAFE
Significant SNPs
Suggestive significant SNPs
-log10PGWAS
0
1
2
3
4
5
6
7
Chr
1
2
3
4
5
7
9
12
17
23
32

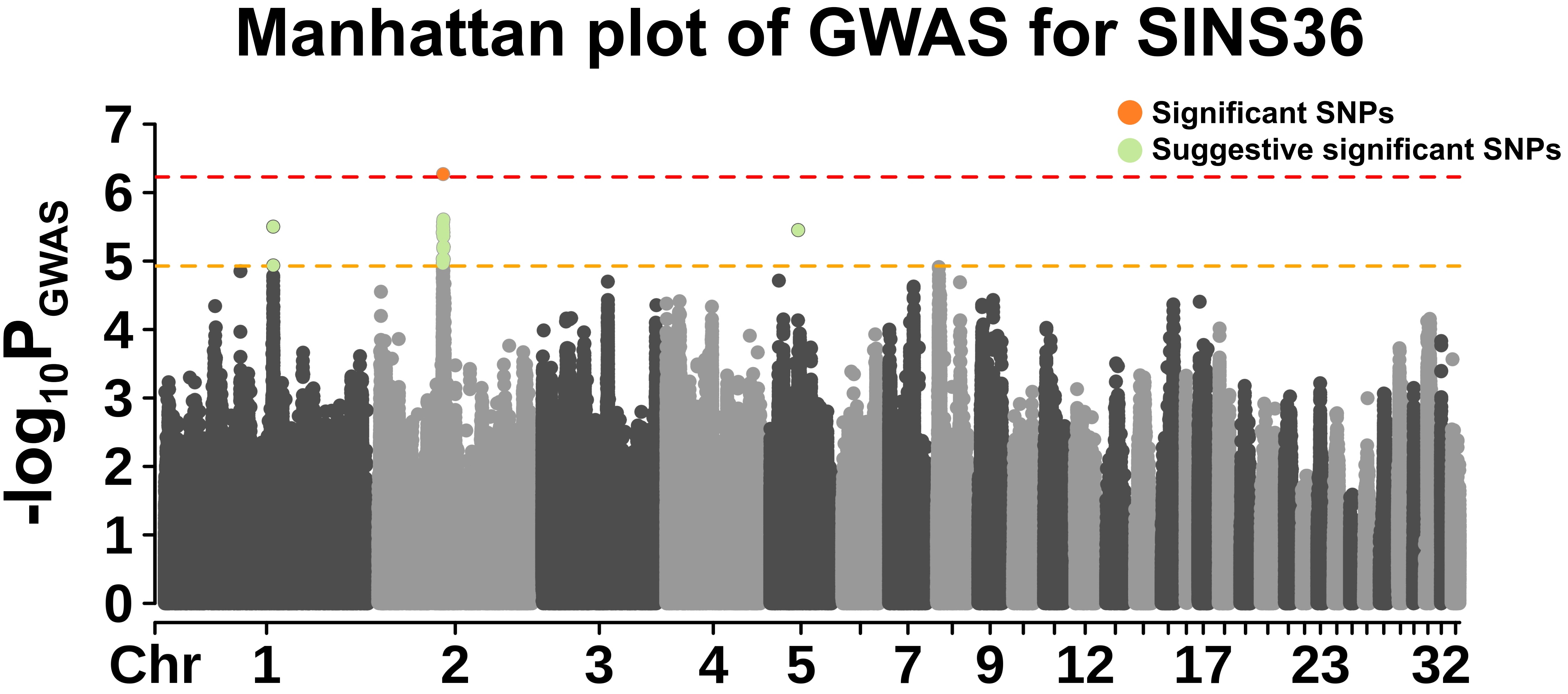

Manhattan plot of GWAS for SINS36
Significant SNPs
Suggestive significant SNPs
$-\log_{10}P_{GWAS}$
0
1
2
3
4
5
6
7
Chr
1
2
3
4
5
7
9
12
17
23
32

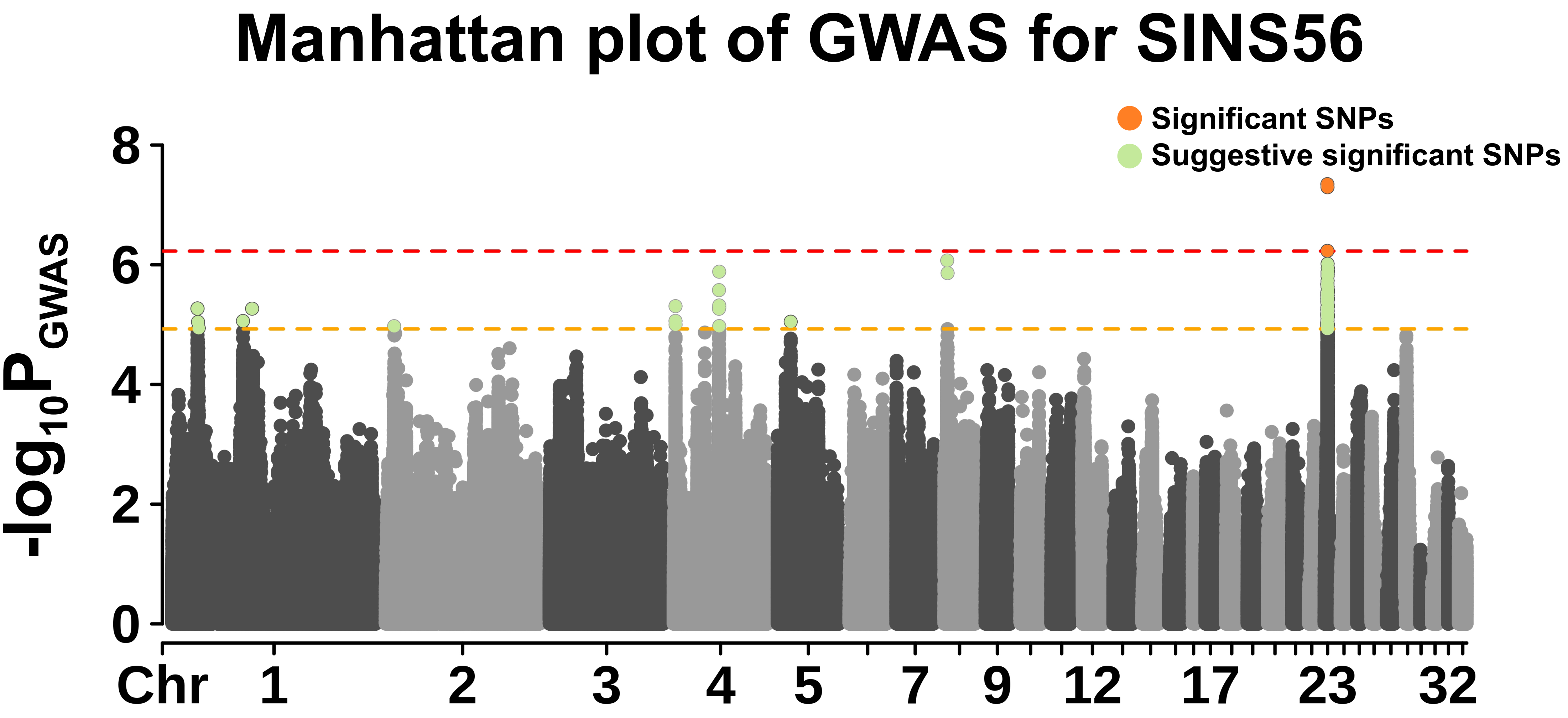
Manhattan plot of GWAS for SINS56
Significant SNPs
Suggestive significant SNPs
$-log_{10}P_{GWAS}$
8
6
4
2
0
Chr
1
2
3
4
5
7
9
12
17
23
32

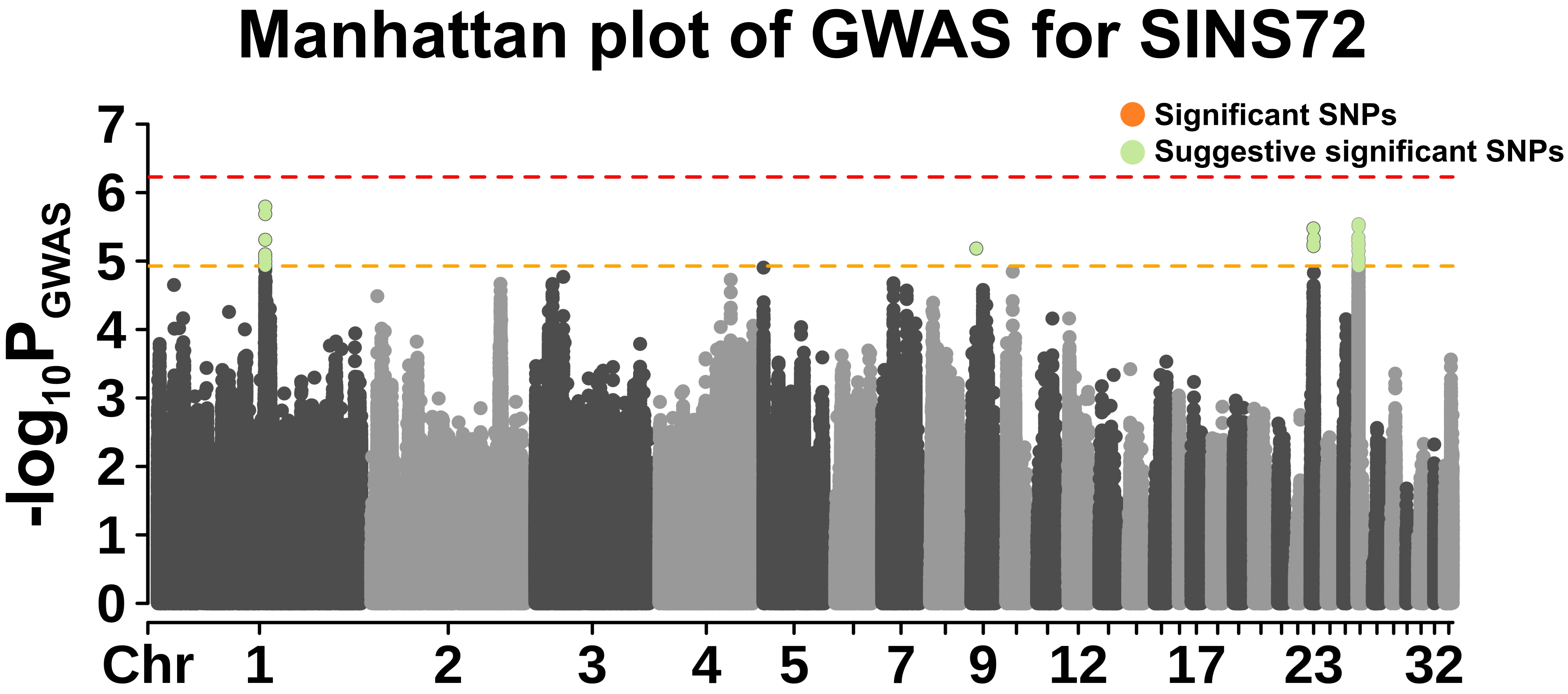

Manhattan plot of GWAS for SINS72
Significant SNPs
Suggestive significant SNPs
-log10PGWAS
0
1
2
3
4
5
6
7
Chr
1
2
3
4
5
7
9
12
17
23
32

Manhattan plot of GWAS for SINS80

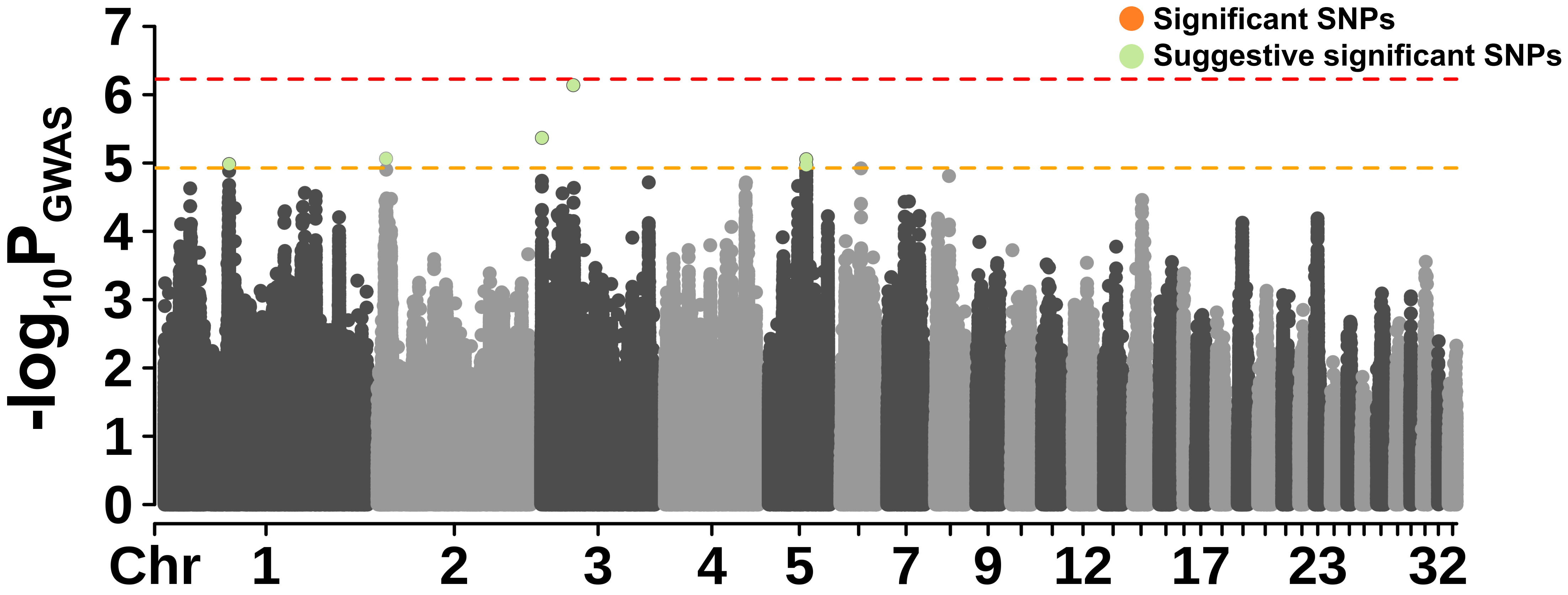

Supplement: Supplementary file 4 — Supplementary Material 4. [file 12864_2024_10551_MOESM4_ESM.pdf]
